# Optimal sampling frequency and site selection for wastewater and environmental surveillance of infectious pathogens: A value of information assessment

**DOI:** 10.1371/journal.pcbi.1013190

**Published:** 2025-06-25

**Authors:** Isabella Impalli, Erik Bergland, Chadi M. Saad-Roy, Bryan T. Grenfell, Simon A. Levin, D. G. Joakim Larsson, Ramanan Laxminarayan

**Affiliations:** 1 One Health Trust, Washington, DC, United States of America; 2 Miller Institute for Basic Research in Science, University of California, Berkeley, California, United States of America; 3 Department of Integrative Biology, University of California, Berkeley, California, United States of America; 4 Department of Ecology and Evolutionary Biology, Princeton University, Princeton, New Jersey, United States of America; 5 School of Public and International Affairs, Princeton University, Princeton, New Jersey, United States of America; 6 High Meadows Environmental Institute, Princeton University, Princeton, New Jersey, United States of America; 7 Department of Infectious Diseases, University of Gothenburg, Gothenburg, Sweden; 8 Centre for Antibiotic Resistance Research in Gothenburg, University of Gothenburg, Gothenburg, Sweden; Dartmouth College, UNITED STATES OF AMERICA

## Abstract

Wastewater and environmental surveillance (WES) is a promising method of detecting infectious diseases in human and animal populations and offers significant advantages over traditional surveillance methods in the early detection of outbreaks. However, WES involves financial and human resources, and public policy decisions must determine whether the benefits of WES outweigh the costs, particularly in low-resource areas. The selection of surveillance sites, sampling frequency, and test sensitivity and specificity are crucial determinants of WES effectiveness and cost-efficiency. We created an analytical model and numerical simulations of disease arrival, spread, and WES strategies to determine the optimal sampling frequency for two interacting patches, each represented by a different sampling site. We show that it is optimal to test in one patch more frequently than it is to test in both patches less frequently if the patches are sufficiently interactive, surveillance is of sufficient sensitivity and specificity, and setup costs are substantial. In our value of information (VOI) assessment, the net value of surveillance information for both patches is non-monotonic with respect to the degree of patch interaction. Increased mixing between the patches allows for quicker surveillance detection but is worse for overall infection burden. Overall, optimizing the value of surveillance information for all patches being surveilled requires coordination and deliberate selection of surveillance sites and sampling frequencies. This paper provides a VOI assessment of WES to determine the optimal number of sites and sampling frequency at a high level of abstraction, leaving opportunity to adapt the model to specific pathogens and populations as needed. Our findings can inform the cost-efficient implementation of WES for infectious diseases, particularly in resource-constrained settings.

## Author summary

For decades, wastewater and environmental surveillance (WES) has helped public health officials track and eradicate poliovirus and recently, WES has shown promise for other diseases. Implementing WES in low-resource settings is sensitive to the balance between the benefits of WES—such as early detection of outbreaks—and the monetary and human resource-related costs. Implicit in this question is the value of the specific attributes of WES system design, including sampling frequency, sample site distribution, and surveillance sensitivity and specificity. In this paper, we use a mathematical model of two interacting subpopulations, or “patches,” to gain high-level insight on how often and where we must conduct WES to ensure an optimized system. We find that it may be optimal to conduct WES in only one of the patches if the two patches are highly interactive and the risk of false positives is low. We also observe that higher rates of disease spread lead to faster disease detection but more cases overall. Our model could be generalized to multiple patches and tailored to specific diseases. Our findings have implications for public health officials who are seeking to implement WES while optimizing use of limited resources.

## Introduction

Wastewater and environmental surveillance (WES) has been shown to be a scalable, effective tool to identify and mitigate the spread of infectious diseases [1]. WES involves sampling the environment directly (e.g., wastewater, surface waters, soil samples) instead of testing individuals for pathogen presence and/or levels [2]. In particular, sampling wastewater is an efficient manner of obtaining a random sample of an entire community in real time [3]. Since the 1970s, WES of poliovirus has helped polio control programs by informing immunization response [2]. WES has been shown to correlate with clinical disease incidence for several viral and bacterial pathogens that spread through varied routes of transmission, including SARS-CoV-2, mpox virus, *Escherichia coli*, influenza virus, and others [4–9], indicating further potential to support clinical or event-based surveillance.

Though WES is subject to limitations, such as being unable to pinpoint identifying characteristics of the infected individuals (which makes contact-tracing efforts, for example, not possible), it offers significant advantages over clinical or event-based surveillance. WES can capture the presence of asymptomatic infections by serving as an indicator of pathogen presence—and sometimes, pathogen levels—that is independent of treatment-seeking behavior or quality of healthcare access [2]. Furthermore, for pathogens such as poliovirus and SARS-CoV-2 [10–12], the early warning capability of WES has been demonstrated, allowing for public health intervention that could avert or mitigate outbreaks.

There is a tradeoff inherent in conducting any disease surveillance, whether WES or clinical surveillance. Resources (e.g., capital, manpower) allocated towards surveillance carry an opportunity cost, since they could have been put towards interventions which directly reduce disease burden [13]. In low- and middle-income country (LMIC) settings, where resources are already stretched thin, it is even more important to ensure that resource allocation is cost-effective and, moreover, that WES system design is as cost-efficient as possible. To this end, methodologies are needed to appropriately evaluate and optimally design surveillance systems.

When designing a WES system, selecting the sites where samples will be taken and determining how often samples will be taken are of critical importance [14,15]. It is expensive to sample too frequently, but sampling too infrequently may miss an outbreak, thereby leading to high levels of infection and associated costs. Similarly, there are tradeoffs in site selection: sampling in too many places is costly, but sampling in too few may miss an outbreak. When multiple populations (e.g., several cities) are connected through travel, tourism, or commuting, disease can spread from one population to another. It follows that surveillance information could be shared between groups, and it may not be necessary to test in all sites. Pinpointing the optimal site selection, as well as the sampling frequency at each of those sites, is central to determining the value of WES.

Value of information (VOI) is an appropriate concept to apply when evaluating WES and optimizing system design [13]. VOI is a statistical and economic concept used to describe the value of additional information in reducing uncertainty and improving decision-making [16]. VOI helps to determine whether the cost of obtaining more information is worth the improved decisions that may result. In this situation, VOI analysis helps decision-makers determine if the information from WES would improve public health decision-making.

Previous studies have considered aspects of valuating WES (e.g., [17–19]), though literature reviews have indicated that generalized and even pathogen-specific guidance on sampling site selection and frequency is lacking [14,20]. Wang and colleagues assessed optimal WES sampling frequency and location for Salmonella in relation to pathogen shedding, sewer connectivity, and test sensitivity in India but did not include the costs associated with the surveillance [15]. A recent study by Olejarz and colleagues found a sampling frequency for environmental surveillance at a single site which optimizes the tradeoff between costs of infection and surveillance [21]. Cheng and colleagues created a simulation-based model that optimizes surveillance according to multiple parameters and objectives [22]. However, a high-level understanding of system design that balances both the number of sites and sampling frequency remains a crucial gap in the literature. In this paper, we developed a generalized mathematical model to optimize surveillance site selection and sampling frequency selection and perform a VOI assessment of the system.

## Methods

To allocate resources for WES, we computed an analytical cost function for a surveillance program based on the frequency of disease arrivals, the disease dynamics, and the frequency of sampling and pathogen detection. We then used computer simulations to overcome some limits of our analytical model. Using these as inputs, we calculated the expected value of information associated with various WES strategies.

### Mathematical model

#### Cost functions.

We minimized the sum of the costs for each subpopulation, or “patch,” with each patch being associated with a single sampling site. The costs incurred by a single patch are comprised of those associated with WES, with disease from an outbreak, and with false positive detection by the surveillance system, given in Equation 1.


$${TC}_{i}=c_{WES,i}+c_{D,i}+c_{S,i}$$ (1)

where TC_*i*_ represents the total costs to patch *i*, *c*_*WES*_ are the costs of WES, *c*_*D*_ are the costs of treating the outbreak, and *c*_*S*_ are the costs of premature public health intervention to “shut down” activity in the patch (i.e., due to a false detection). While we acknowledge that all shutdowns would have a cost, it is the cost of premature ones that influence decision-making. Incorporating a blanket shut down cost is akin to introducing a constant into the model, since the patch always shuts down eventually. We therefore opt to only account for the premature shutdown cost in this model.

The cost of WES has two elements: a fixed cost to set up the program and a variable cost that is a function of the frequency of WES sampling. For a given patch, the setup cost is zero if we choose not to establish WES at that patch. We assume that when the pathogen is detected, immediate action is taken to stop further disease spread, and therefore, the time of detection applies to both patches. The costs associated with disease are proportional to the number of infections which make up the outbreak at the time of detection.

The global total cost, or the total cost to both patches, is the sum of the local total costs to each patch *i* and is given by Equation 2.


$$\begin{array}{c} TC=\sum_{i}k_{i}\cdot 1_{\left\{f_{i}>0\right\}}+a_{i}f_{i}{E[t}_{detection}]+C_{I,i}𝔼{[I}_{detection,i}]+\;C_{S,i}N_{i}P(\text{false detection}) \end{array}$$ (2)

where *k*_*i*_ is the fixed cost of setting up WES in patch *i*, *f*_*i*_ is the frequency of sampling in patch *i*, *a*_*i*_ is the cost per sampling day in patch *i*, *t*_*detection*_ is the total time over which samples are being taken, *C*_*I,i*_ is the cost associated with each infection in patch *i*, *I*_*detection,i*_ is the number of infections in patch *i* at the expected time of outbreak detection by WES, *C*_*S,i*_ is the per capita cost associated with shutting down due to a false positive detection in patch *i*, *N*_*i*_ is the population of patch *i*, □[X] is the expectation or average value of *X,* and ℙ[X] is the probability of *X*.

To operationalize the model in specific contexts, the cost of infection and premature shutdown as well as the costs associated with setting up and conducting WES can be assigned appropriate values from the literature. In our model, we have a false positive shutdown cost that scales with the population size. To calculate the expected detection time and number of cases for each patch given WES, we consider the pathogen arrivals, dynamics, and detection methodology.

#### Pathogen arrivals and dynamics.

In general, we suppose that the model of the epidemic can be broken down into two parts: a random, discrete jump in the number of infections due to pathogen arrival in a patch, and growth in the number of infections resulting from the initial arrival event. Surveillance takes place independently of this process. We assume that once a sample detects the pathogen, there is immediate and successful shutdown of all patches, halting growth. For this reason, we consider only exponential growth in the number of infections by linearizing a compartmental model about a disease-free state (see Appendix A in S1 Text for the derivation of disease dynamics), with no higher-order terms to cause the model to reach equilibrium.

The patches have some migration between them that makes it possible for disease to spread from one subpopulation to the others. The length of time before the infection arrives in each patch is modeled by an exponential random variable with its own rate, which represents the risk each subpopulation faces. The minimum of these arrival times is treated as the onset of the outbreak and determines the patch of origin. Once the disease arrives, the epidemic can spread to additional subpopulations. The number of infections in patch *i*, denoted *I*_*i*_, evolves according to Equation 3.


$$\frac{dI_{i}}{dt}=r_{i}I_{i}+\sum_{j,j\neq i\;}I_{j}\eta_{j\to i}$$ (3)

where *I*_*i*_ represents the number of infections in patch *i* at time *t, r*_*i*_ represents the effective growth rate of infections within patch *i,* and *η*_*j→i*_ represents the rate at which infected individuals from patch *j* contribute to the growth of infections in patch *i*.

#### Detection of epidemics, analytical model.

When the epidemic begins in a patch, we suppose that the length of time until it is detected in another patch can be modeled by an exponential random variable. The detection time of the epidemic is then given by the first detection time across all patches. In Appendix B in S1 Text, we demonstrate that the minimum of a group of independent exponential random variables is once again an exponential random variable. Therefore, the detection time of the epidemic is also distributed exponentially, with a rate parameter we call the surveillance effectiveness that depends on the patch of origin. For further details on the computation of the surveillance effectiveness, see Appendix C in S1 Text.

#### Detection of epidemics, numerical simulations.

The probability of detecting an epidemic discussed in the previous section is independent of the number of infected individuals. This restriction is in line with the analysis given in Olejarz and colleagues [21], where detection events occurred with a fixed probability. Ultimately, this choice comes down to the tractability of the mathematics: introducing a dependence on the number of infected individuals improves the realism of the model, but analytical results are often difficult or impossible to obtain.

To incorporate the dependence, we ran numerical simulations of the epidemic. Every quarter day for 100 days, the fraction of infected individuals was computed, which served as the probability that a WES sample taken at that time truly contained the pathogen of interest. Prior to the onset of infections, samples could return a false positive with a given probability. Ten independent Bernoulli trials were conducted for each patch—meant to represent ten independent environmental samples—and if any were successful, a shutdown was triggered. We then investigated scenarios where a certain threshold of the ten samples must be positive before shutdown was triggered.

After running 100,000 simulations for a given set of parameter values, every possible pair of testing periods from a preselected list was used to examine the resulting potential detections. As soon as a potential detection event in a patch coincided with a testing day in the same patch indicated by the protocol, the epidemic is considered detected under the chosen protocol and the duration and size of the outbreak are recorded. If no detections occurred on indicated testing days for the entire duration of the simulation, the detection time was recorded as 100 days, and the outbreak size was set to the maximum population of both patches, and we assume the patches were shut down at that point. The expected length of the epidemic and the expected number of infected individuals were estimated from the sample mean of those quantities, which then served as replacements for the Equations 4 and 5, presented in the Results section. Simulations were limited to two patches for both computational efficiency and interpretability. Testing periods for the two patches consisted of all pairwise combinations of: 0.25, 0.5, 1, 2, 3, 4, 5, 6, 7, 8, 9, 10, 11, 12, 13, 14, and infinity (which represented no WES in a patch). Other parameter values tested are indicated in the results.

### Value of information assessment

In general, the value of information, or VOI, can be described as the difference between the outcomes of decisions made under current information and the outcomes of decisions made under additional information that reduces decision uncertainty [23]. We can envision the model presented above as a decision problem where a decision-maker determines when to shut down the patches. At any given point in time, it is uncertain whether the patches should be shut down, but WES could provide some information regarding the state of the system (i.e., an indication of whether infections are present in either patch) that may improve decision-making. In other words, through WES, we seek to reduce the uncertainty around the exact time of the first disease arrival in either patch (*t*_arrival_), which is an exponentially distributed random variable with rate parameter *λ*_tot_. We describe the VOI from WES in terms of reduction in expected loss over the course of the simulation.

Our VOI framework is derived in the extended methods of Appendix D in S1 Text, where we compute several metrics common to VOI analysis: the expected value of perfect information (EVPI), the expected value of sample information (EVSI), and the expected net gain of sampling (ENGS). EVPI reflects the value of resolving all uncertainty in a decision model, EVSI reflects the value of reducing uncertainty in a decision model, and ENGS is the EVSI minus the costs of collecting the information [23]. We define the EVSI and ENGS for a particular surveillance strategy *x* (e.g., testing daily in patch 1/weekly in patch 2) as EVSI_*x*_ and ENGS_*x*_. We note that in this model, our counterfactual policy of no surveillance necessarily leads to disease taking over the entire patch prior to shutdown; we also explore the possibility of a policy which defaults to shutdown at □[*t*_arrival_] in Appendix D in S1 Text. As it pertains to the Main Text, we employ the VOI concept mainly to help assess and compare different WES strategies which provide information on *t*_arrival_.

## Results

### Analytical model

Given that the outbreak spreads according to Equation 3 and with arrival and detection as prescribed in the Methods section, we compute the expected time to detection (*t*_*detection*_) and expected outbreak size (*I*_*detection*_) analytically. The details are given in Appendix E in S1 Text. The resulting values are summarized in Equations 4 and 5.


$$𝔼\left[t_{detection}\right]=\frac{n+1_{{⋃}_{j=1}^{n}\left\{\rho_{j}>0\right\}}+\sum_{i=1}^{n}\frac{\lambda_{i}}{s_{i}}}{\lambda_{tot}+\sum_{i=1}^{n}\rho_{i}f_{i}}$$ (4)

$$E[I_{detection}]=\frac{\sum_{i=1}^{n}{\lambda_{i}\left(Id-\frac{A}{s_{i}}\right)}^{-1}e_{i}}{\lambda_{tot}+\sum_{i=1}^{n}\rho_{i}f_{i}}$$ (5)

where *n* is the total number of patches, *ρ*_*i*_ is the rate of false positives per test day in a patch, *λ*_*tot*_ is the sum of the arrival rates *λ*_*i*_ over all patches, *Id* is the identity matrix, *A* is the matrix which describes the rate of disease growth in each patch and relationships between patches determined by Equation 3, *s*_*i*_ is the surveillance effectiveness when the disease begins in patch *i*, *f*_*i*_ is the frequency of testing in patch *i*, and **e**_***i***_ is a vector which indicates which patch is being discussed.

By substituting Equations 4 and 5 into Equation 2, we obtain the total cost function in Equation 6.


$$TC=\sum_{i=1}^{n}\left(k_{i}\cdot 1_{\left\{f_{i}>0\right\}}+a_{i}f_{i}\left(\frac{n+1_{{⋃}_{j=1}^{n}\left\{\rho_{j}>0\right\}}+\sum_{j=1}^{n}\frac{\lambda_{j}}{s_{j}}}{\lambda_{tot}+\sum_{j=1}^{n}\rho_{j}f_{j}}\right)+\frac{\lambda_{i}}{\lambda_{tot}+\sum_{j=1}^{n}\rho_{j}f_{j}}\left[{{C_{I}}^{T}\left(Id-\frac{A}{s_{i}}\right)}^{-1}{\text{e}}_{i}\right]+\frac{C_{S,i}N_{i}\sum_{j=1}^{n}\rho_{j}f_{j}}{\lambda_{tot}+\sum_{j=1}^{n}\rho_{j}f_{j}}\right)$$ (6)

Where *TC* is total costs, *k* is the vector of WES setup costs, *a* is the vector of costs per sample, *f* is the vector of sampling frequencies, *C*_*I*_ is the vector whose elements are the cost per infection in each patch, *C*_*S*_ is the vector whose elements are the per capita cost of premature shutdown in each patch, and *N* is vector of the populations of the patches. Equation 6 can then be minimized to yield the optimal surveillance strategy for a given set of parameter values.

Under all parameter values considered, these minima consist of nonzero frequencies for all patches, indicating that no patch benefits from the option to simply forego setting up a surveillance program. We also calculate the best response curves for each patch. The best response curve indicates the frequency of WES for the patch which minimizes the local total costs to the patch, given some WES frequency for the other patch.

**Fig 1:**
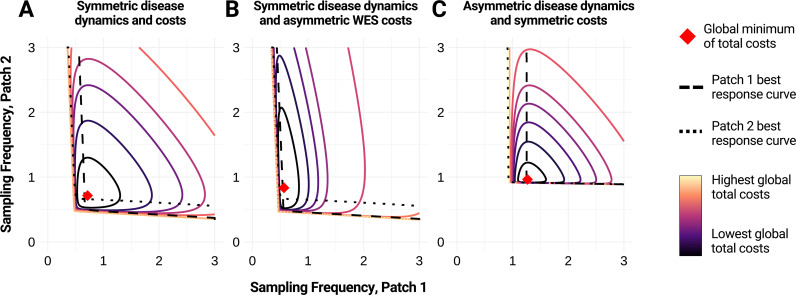
Optimal surveillance strategies for two-patch system from analytical model. We illustrate the global total costs (colored gradient) associated with various wastewater and environmental surveillance (WES) strategies for different parameter sets that reflect A) symmetric disease dynamics and costs, B) symmetric disease dynamics and asymmetric WES costs, and C) asymmetric disease dynamics and symmetric costs. The red diamond represents the global minimum of total costs for each parameter set. Best response curves for patch 1 are shown as a dashed black line; best response curves for patch 2 are shown as a dotted black line. Parameter values: A) *λ*_*1*_ = *λ*_*2 *_= 0.15, *r*_*1*_ = *r*_*2*_* *= 0.2, *η*_*2→1*_* = η*_*1→2 *_= 0.01, *k*_*1*_* = k*_*2*_* *= 50, *a*_*1*_* = a*_*2*_ = 50, *C*_*I,1*_ = *C*_*I,2*_ = 10, *ρ*_*1*_* = ρ*_*2*_ = 0; B) same as A) except *a*_*1*_ = 200; C) same as A) except *r*_*1*_ = 0.4 and *η*_*2→1*_* = η*_*1→2 *_= 0.003. Based on Equation 6.

Contour plots show a concentric, regular pattern around the optimal strategy (Fig 1). When all parameter values between the two patches are the same, the global total cost function is symmetric about *f*_*2*_ = *f*_*1*_, and the response curves are mirrored over *f*_*2*_ = *f*_*1*_ (Fig 1A). Sufficiently small testing frequencies result in infinite cost due to disease burden, since the resulting distribution of infected individuals at detection has an infinite expectation value due to runaway outbreaks. As such, we do not plot the response curves in this regime. For more details on this phenomenon, see Appendix E in S1 Text.

If the patches are asymmetric in either their costs (Fig 1B) or disease dynamics (Fig 1C), an optimal WES strategy emerges that involves sampling each site at a different frequency. While the analytical model does capture key qualitative aspects of the surveillance problem, its shortcomings (including infinite costs for some sampling strategies and complete independence of the number of infected individuals) indicate it is best used in conjunction with more general numerical simulations, which are presented below.

### Simulated two-patch dynamics

We simulate random disease arrival described by a Poisson process and spread according to Equation 3. We overlay a WES protocol which takes ten independent samples. Each sample has a probability of detection that varies with level of disease presence (see Methods). Initially, we do not allow for false positives.

**Fig 2:**
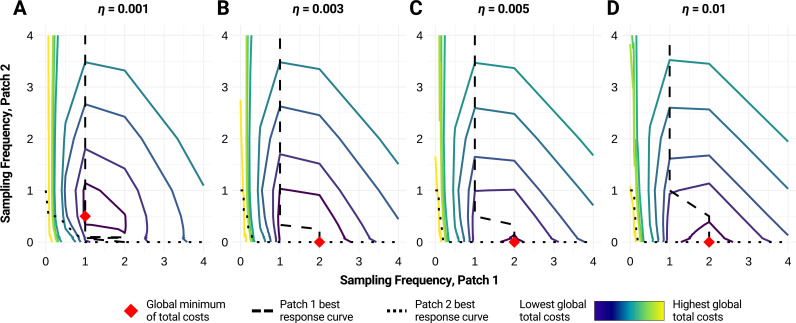
Optimal strategies by level of interaction in patches with asymmetric disease dynamics. We illustrate the global total costs (colored gradient) associated with various WES strategies when A) *η* = 0.001, B) *η* = 0.003, C) *η* = 0.005, and D) *η* = 0.01, where *η = η*_*1→2*_* *= *η*_*2→1*_ is the strength of interaction between patches. The red diamond represents the global minimum of total costs for each parameter set. Best response curves for patch 1 are shown as a dashed black line; best response curves for patch 2 are shown as a dotted black line. Based on 100,000 simulations and a WES protocol that takes 10 independent samples at specified frequencies (see Methods); the gradient in between simulated points is interpolated. Parameter values: patch size = 5000, *λ*_*1*_ = *λ*_*2 *_= 0.15, *r*_*1*_ = 0.4, *r*_*2*_* *= 0.2, *k*_*1*_* = k*_*2*_* *= 50, *a*_*1*_* = a*_*2*_ = 50, *C*_*I,1*_ = *C*_*I,2*_ = 10, false detection probability = 0.

As with the analytical model, contour plots of global total costs show an interpolated concentric pattern around the global minimum. If patches have asymmetric disease dynamics, we can forgo sampling in the patch with lower rate of spread (*r*) if patch interaction (*η*_*1→2*_* *= *η*_*2→1*_) is strong enough (Fig 2). Even in such cases where the globally optimal strategy remains the same, the total cost gradient continues to shift around the minima. If setup costs are large enough in both patches, the globally optimal strategy favors the switch from testing in two patches to one patch at lower levels of interaction (Fig A in S1 Text). It is also possible for setup costs in a patch (or both patches) to be prohibitively high, thereby precluding surveillance in that patch (or both patches), regardless of any other disease or interaction dynamics.

When disease dynamics are symmetric, a noteworthy phenomenon emerges as patch interaction increases. At lower levels of interaction, if sampling frequencies are different between the two patches, we observe that the expected size of the outbreak upon detection is different in each patch; testing more frequently in a patch results in less cases in that patch. However, at high levels of interaction, we observe that the size of the outbreaks upon detection is approximately the same in both patches even when sampling frequencies differ (for more on the mathematical underpinnings of this phenomenon, see Appendix F in S1 Text). For example, if testing is conducted daily in patch 1/weekly in patch 2, we may observe that patch 1 and patch 2 both have 50 cases upon detection. Moreover, the expected size of the outbreak in patch 1 and patch 2 would be 50 cases each, regardless of whether testing is conducted daily in patch 1/weekly in patch 2 or weekly in patch 1/daily in patch 2.

**Fig 3:**
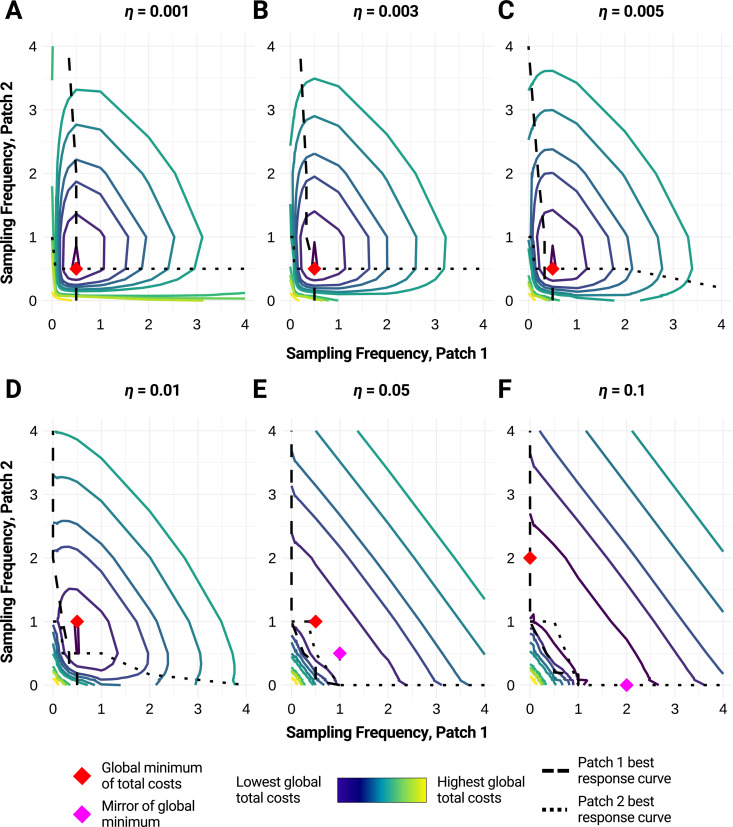
Optimal strategies by level of interaction in patches with asymmetric disease costs. We illustrate the global total costs (colored gradient) associated with various WES strategies when A) *η* = 0.001, B) *η* = 0.003, C) *η* = 0.005, D) *η* = 0.01, E) *η* = 0.05, and F) *η* = 0.1, where *η = η*_*1→2*_* *= *η*_*2→1*_ is the strength of interaction between patches. The red diamond represents the simulated global minimum of total costs for each parameter set. The pink diamond represents the strategy with the second-lowest total costs for each parameter set if that strategy is a mirror of the simulated minimum. Best response curves for patch 1 are shown as a dashed black line; best response curves for patch 2 are shown as a dotted black line. Based on 100,000 simulations and a WES protocol that takes 10 independent samples at specified frequencies (see Methods); the gradient in between simulated points is interpolated. Parameter values: patch size = 5000, *λ*_*1*_ = *λ*_*2 *_= 0.15, *r*_*1*_ = *r*_*2*_* *= 0.2, *k*_*1*_* = k*_*2*_* *= 50, *a*_*1*_* = a*_*2*_ = 50, *C*_*I,1*_ = 5, *C*_*I,2*_ = 10, false detection probability = 0.

This forced symmetry in outbreak size has consequences for the total cost function. When the costs of the disease differ but interaction is high and disease dynamics are symmetric, multiple optimal strategies can emerge. In Appendix G in S1 Text, we show that when the average case count in both patches are equal, one can swap the sampling frequencies of the patches without changing the total cost. In our simulations, we observe one true optimal strategy, with the mirror strategy (patch frequencies interchanged) being the close second-best strategy at sufficiently high levels of interaction (Fig 3). For the parameter set simulated, we see a switch to single patch testing just as in the case with asymmetric disease dynamics (Fig 2). However, unlike the case with asymmetric disease dynamics, it is likely just as optimal to surveil only patch 1 as it is to surveil only patch 2 (Fig 3F).

The ability to interchange the surveillance frequencies of the patches is not seen if the costs associated with WES are asymmetric (also see Appendix G in S1 Text). If costs to conduct surveillance in one patch are high enough, *ceteris paribus*, the optimal strategy will shift to single-patch testing in the patch with lower surveillance costs as interaction increases, and the mirror strategy will not be a second optimum (Fig B in S1 Text). There can also be cases where the costs of WES are so high that it is most cost-efficient to forgo testing in both patches and incur costs associated with widespread disease. The best response curves trend towards lower frequencies as connectivity increases (Fig B in S1 Text).

In all cases of asymmetry—disease dynamics, disease costs, or surveillance costs—we note that once the optimal strategy resorts to single-patch testing, the optimal frequency of sampling at that single patch is higher than the individual patch sampling frequencies at the optima of lower levels of patch interaction.

### False positives and shutdown thresholds

**Fig 4:**
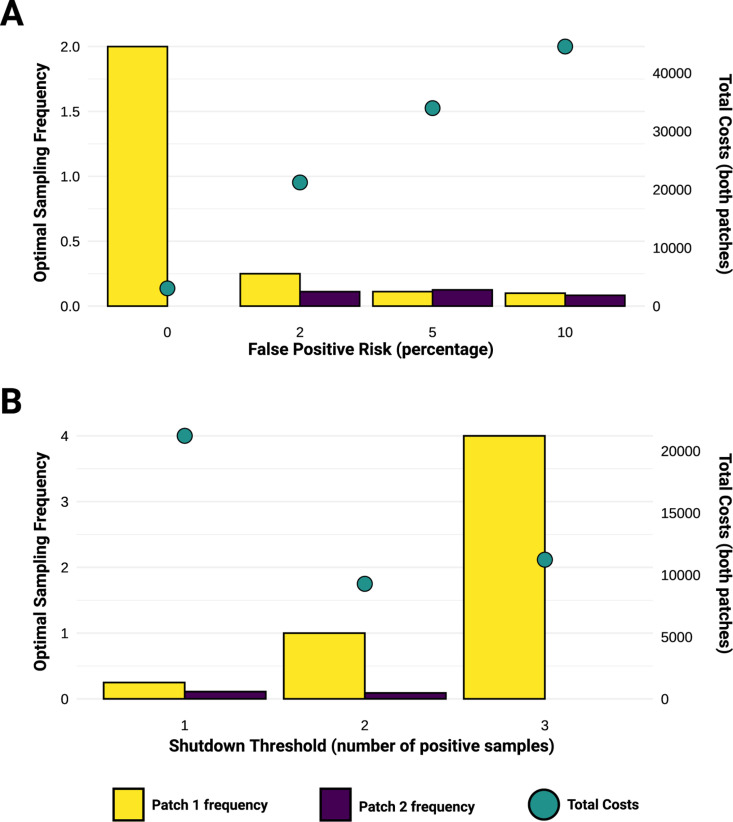
Impact of false positivity and differing shutdown thresholds on optimal strategy. We illustrate the optimal sampling frequency in each patch and total costs associated with A) increasing levels of WES false positive risk and B) an increasing shutdown threshold under a constant false positive risk of 2%. Other parameter values: patch size = 5000, *λ*_*1*_ = *λ*_*2 *_= 0.15, *r*_*1*_ = 0.4, *r*_*2*_* *= 0.2, *η*_*2→1*_* = η*_*1→2 *_= 0.01, *k*_*1*_* = k*_*2*_* *= 50, *a*_*1*_* = a*_*2*_ = 50, *C*_*I,1*_ = *C*_*I,2*_ = 10, *C*_*S,1*_ = *C*_*S,2*_ = 5.

In all results presented up to this point, we have assumed that the probability of false positives—and thus, premature and unnecessary patch shutdowns—was zero. By increasing this probability to two percent in a scenario with asymmetric growth rates, we see that one-patch testing is no longer the optimal strategy. Both patches once again optimally conduct WES, and the total costs are higher (Fig 4A). Importantly, while total costs increase with false positivity, the optimal frequency in both patches does not decrease as steadily. Under the parameter set shown, we observe a decrease across both patches, though in view of a single patch, the frequency does not necessarily decrease much as false positivity increases (Fig 4A).

The costs of a premature shutdown can be counteracted by modifying the detection threshold. Previously, we assumed that only one of the ten independent WES samples should be positive to trigger the shutdown. By requiring more positive tests surveillance gains utility, as evidenced by an initial decrease in total costs and an increase in testing frequency (Fig 4B). However, as we move from requiring two to three positive samples under the parameter set shown, total costs rise again (Fig 4B). While a different parameter set may yield decreasing total costs at the same threshold (Fig C in S1 Text), we expect that at some higher threshold.

Whether the increased shutdown threshold enforces a return to two-patch testing depends on the other parameters; testing in both patches can sometimes be justified once the shutdown threshold increases (Fig C, panel B in S1 Text). We note that if there are sufficiently high premature shutdown costs and false positive rates (i.e., the surveillance has low sensitivity), then the optimal strategy is to forgo surveillance entirely (Fig D in S1 Text). If we assume that ‘better’ surveillance (i.e., a lower false positive rate) is also more expensive, then these tradeoffs could be represented explicitly in the model but would be dependent on the parameter values chosen.

### Decision-making and value of information

We also calculate the value of information local to each patch as well as globally for both patches, mainly focusing on scenarios where the probability of a false positive is zero. Given the sampling frequency of the other patch, the EVSI for a given patch increases with sampling frequency and approaches the EVPI local to the patch (Fig E, panel A in S1 Text). Local WES costs increase with frequency of sampling over a given simulation period. The local maxima of ENGS, which is given by ENGS along the best response curve for the patch, increase as the sampling frequency increases in the other patch (Fig E, panel B in S1 Text). The local ENGS for the patch peaks at the same frequency that local total costs are minimized (Fig F in S1 Text).

**Fig 5:**
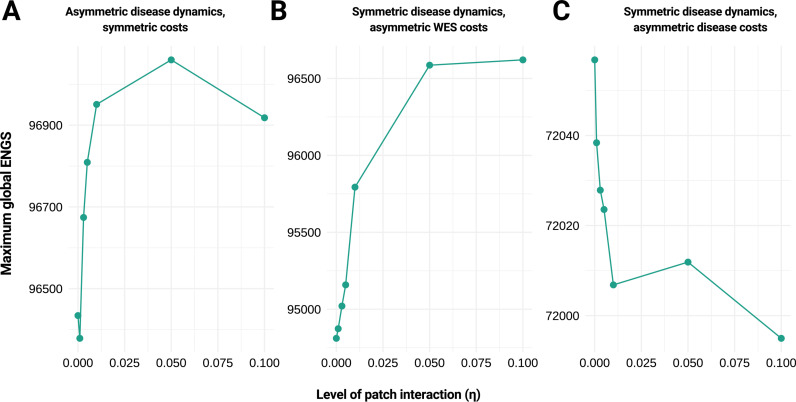
Maximum global expected net gain of sampling. We illustrate the maximum global expected net gain of sampling (ENGS) associated with various wastewater and environmental surveillance (WES) strategies for different parameter sets that reflect A) asymmetric disease dynamics and symmetric costs, B) symmetric disease dynamics and asymmetric WES costs, and C) symmetric disease dynamics and asymmetric disease costs. Based on 100,000 simulations and a WES protocol that takes 10 independent samples at specified frequencies with a detection rate dependent on infection burden (see Methods). Data points are connected by straight lines for illustration. Parameter values: A) *λ*_*1*_ = *λ*_*2 *_= 0.15, *r*_*1*_ = 0.4, *r*_*2*_* *= 0.2, *η*_*2→1*_* = η*_*1→2 *_= 0.01, *k*_*1*_* = k*_*2*_* *= 50, *a*_*1*_* = a*_*2*_ = 50, *C*_*I,1*_ = *C*_*I,2*_ = 10, false detection probability = 0, B) same as A) except *r*_*1*_* *= 0.2 and *a*_*1*_ = 200; C) same as A) except *r*_*1*_* *= 0.2 and *C*_*I,1*_ = 5.

The maximum global ENGS does not exhibit monotonic behavior with respect to the level of patch interaction, even as the global strategy remains the same (Fig 5). It is not possible to predict whether the ENGS will increase or decrease as the patches become increasingly connected. While the time of detection decreases as the patches become increasingly connected (Fig G, panel A in S1 Text), the total size of the outbreak upon detection in both patches decreases before rising again as connection increases (Fig G, panel B in S1 Text). The forced symmetry seen in Fig 3 is demonstrated explicitly in Fig G, panel C in S1 Text. As the patches become increasingly interactive, the average of the absolute value of the difference between the number of cases in patches one and two upon detection approaches zero.

Lastly, our model is designed under the premise that a single decision-maker is responsible for selecting which patches to sample and at which frequencies. However, we may consider a situation where each patch is independently operating its own surveillance system. We can calculate the expected net loss associated with suboptimal WES strategies, which may occur if the patches do not coordinate. Of particular interest is the net losses incurred at the Nash equilibria (NE), which is the potentially suboptimal WES strategy which occurs when the patches do not coordinate. NE are stable strategies and will not change unless there is an authority forcing the patches to operate at the globally optimal strategy. At the discrete frequencies simulated, Δ_ENGS,NE_ does not have a predictable association with the level of patch interaction. In some cases, the NE matches the globally optimal strategy as patch interaction increases (Fig H, panel A in S1 Text), but in others, it does not, and Δ_ENGS,NE_ fluctuates as patch interaction increases (Fig H, panels B and C in S1 Text). At the discrete frequencies simulated, we also observe that the NE can overlap with the globally optimal strategy. When disease dynamics are uncoupled (when *η*_*2→1*_* = η*_*1→2 *_= 0), the NE matches the globally optimal strategy (Δ_ENGS,NE_ = 0) under various parameter sets (Fig I in S1 Text). We note that values of 0 may reflect the discrete frequencies simulated, and it is possible that if we could test frequencies with more granularity that the values may not perfectly overlap.

## Discussion

In this paper, we present an analytical model and accompanying numerical simulations for infectious disease arrival, spread, and WES to find the optimal strategies for site selection and sampling frequency. Though our model is generalizable to *n* patches, we report the complex dynamics observed between *n = *2 patches, and we also use VOI techniques to evaluate the system. While we report general trends, the truly optimal strategy is wholly dependent on the relationship between all parameter values in the model, which would be specific to the context being studied. Thus, we emphasize the importance to practitioners to choose appropriate parameter values, particularly the level of patch interaction. In this study, we focus on changing one parameter at a time to gain intuition on how the optimal surveillance strategy adjusts to different model parameters.

In general, we observe that it is optimal to only test in one of the patches as interaction between the patches increases, with some caveats. Factors that affect the switch to single-patch surveillance include the level of asymmetry in either disease dynamics, disease cost, or the cost of surveillance, as well as the quality of surveillance (i.e., the risk of a false detection). We observe that when the optimal strategy is to only surveil in one patch, the optimal frequency of sampling in the single patch is increased compared to testing both patches; this observation was consistent across different types of asymmetries. To generalize to more than two patches, these results suggest that as subpopulations experience more interaction, it may be more cost-efficient to test at one site more frequently than it is to test at multiple sites less frequently if setup costs are substantial and the risk of false positives is low.

However, if surveillance is of insufficient quality (i.e., the false positivity rate is too high), then it may be more cost-efficient to test in multiple sites less frequently, which safeguards against false positives. Another method of safeguarding against false positives is to increase the threshold of positive WES samples needed to trigger a shutdown, which will reduce total costs at first. However, if we continue to increase that threshold, we create *de facto* false negatives, wherein the pathogen is present in the patch but we do not take action and therefore incur higher infection costs. With full knowledge of other parameters, there is an optimal threshold to counteract each level of potential false positives.

We also find that at high levels of interaction for patches with symmetric disease dynamics, patch sampling frequencies for the optimal strategy could be interchanged to yield a second optimal strategy. The emergence of one true optimal strategy in these cases was likely due to simulation noise. Importantly, we see that this ability to interchange the frequencies held even when testing a single patch. Such cases are important when considering WES in the context of communities with limited wastewater connectivity in resource-constrained areas. For example, the United Nations Statistics Division/United Nations Environment Programme questionnaire on Environment Statistics indicated that in Kenya, only 8.5% of the resident population is connected to a wastewater collecting system as of 2020 [24]. Wastewater management systems such as septic tanks also provide barriers to representative WES systems [2]. Barriers to environmental surveillance in LMIC settings are not just infrastructural; they may be institutional or sociocultural as well [25]. For example, bureaucracy may impede resource and sampling site access, and implementing WES is subject to the skills and training of locals in specific methodologies [25]. Our results indicate that a subpopulation with limited sewage connectivity could still be adequately surveilled through WES if that subpopulation interacts sufficiently with another subpopulation that has sewage connectivity.

Operationalizing our model requires sourcing appropriate values for all parameters. The cost of setting up WES and the cost per sample are highly specific to individual contexts, as are the size and reflectiveness of the sites. Our model assumes that the surveillance sites are predefined and are perfectly reflective of the population being surveilled. However, when designing a WES system, choosing the location of surveillance sites is critical. Decision-makers must balance choosing a site that represents a sufficient portion of the population and preserves the integrity of the samples, as the target markers may degrade over time and distance [26].

We find that it is difficult to predict how the values of the global total cost function and ENGS function will shift as we increase interaction between the patches. Increased mixing and disease spread, which result from increasing levels of interaction, contribute to both higher infection burden and earlier WES detection of the outbreak. The maximum ENGS changes non-monotonically as the level of interaction between the patches increases, suggesting that the tradeoff between infection burden and earlier detection times is complex, and more investigation is needed. Similarly, we see unpredictable behavior with respect to the expected net losses of sub-optimal WES strategies.

We also find that the optimal strategy may stay the same for a patch even though the total cost function and the ENGS continue to shift with changes in the level of patch interaction. It is likely that this ‘stickiness’ result from the fact that our model only simulates discrete frequencies of WES sampling; total costs and ENGS shift before the optimal strategy ‘jumps’ as we change the level of interaction. However, preliminary results from our analytical model suggest that when patch interaction is weak it does not strongly affect the optimal sampling strategy, but instead only modifies the total cost of that strategy. For a heuristic argument on why this may be so, see Appendix H in S1 Text. Ultimately, additional research is needed to confirm the persistence of an optimal strategy in the face of changing interaction levels.

The dynamics of our model assume that individuals only migrate from one patch for short periods of time before returning to their original patch. Relaxing this assumption would alter the maximum number of cases in a patch and the success probabilities for tests. The first effect arises because simulations cap infections at the local population size, so significant net migration between patches could impact cost estimates—though such demographic shifts would need to be extreme or sustained to matter, typically occurring on timescales outside the model’s scope. The second effect involves changes to the fraction of infected individuals due to migration, potentially influencing detection probabilities. However, unless the migrating population is large or non-homogeneous (i.e., only healthy or infected), these shifts are unlikely to meaningfully alter the behavior of the model.

A key benefit of this paper is the generalizability of the results. Our mathematical model relies on “patches,” which can represent subpopulations of a single city that are connected to separate municipal sewage lines, a large city and its nearby suburb(s), or cities on different continents. In this paper, we focus on the idea that the WES system operates in an area which is controlled by a single decision-maker whose objective is to minimize total costs for the system. However, our setup allows for generalization to situations where the patches may not cooperate. Through an analysis of the ENGS local to each patch, we find that each patch benefits from increased sampling frequency in the other patch, likely because the increased sampling frequency contributes to lowering the shared time of detection. It is important to understand the differing incentives for each patch when they operate independently. Perhaps an urban city and a nearby suburb are each operating their own surveillance systems, subject to their own political and budgetary constraints. In this respect, our results reproduce many findings from previous analyses of two-patch dynamics. Klepac and colleagues targeted optimal vaccination coverage, not surveillance, though they also observed that in cases with uncoupled disease dynamics the patches achieved a global optimal strategy without any coordination [27]. On the other hand, both Smith and colleagues [28] as well as Drohan and colleagues [29] demonstrated the potential for free-riding behavior in improperly incentivized healthcare facilities. However, unlike all three of these efforts, our model does not assume that the system has reached equilibrium. To the contrary, we focus only on the transient dynamics of a disease outbreak, demonstrating the robustness of the game-theoretic elements to the underlying model of the disease. We also note that the disease dynamics we simulate are robust to model choice, making the model applicable to a wide variety of pathogens (see Appendix A in S1 Text).

In future work, we plan to extend this analysis to more than two patches. In the current study, we assume that immediate action was taken to shut down both patches and halt the spread of disease upon disease detection. However, with more patches, it would be possible to consider more complicated WES and patch shutdown strategies. In this paper, we do not define public health interventions and their associated costs beyond complete patch shutdown. Nascimento de Lima and colleagues conducted a valuation of environmental surveillance for COVID-19 and assumed that there were costs associated with the non-pharmaceutical interventions triggered upon disease detection [17]. We plan to extend our analysis with a more nuanced underlying decision model containing a suite of policy decisions that have different outcomes, costs, and rates of success.

Our model underestimates the value of surveillance information by only accounting for the direct costs associated with the avertable infection burden. However, WES provides information that is not explicitly linked to policy decision-making and can provide insights on viral evolution [30] or seasonal trends [31]. When information is not linked to decision-making, it is challenging to quantify its value [13,17]. VOI frameworks, for example, are built from decision-making models. We also note that our application of VOI in this paper is simplistic because we are only considering one parameter of interest, and our only decision option was to shut down the patches entirely. However, a more complicated decision model as described above would better draw upon the potential of VOI in this context.

In our model, we assume that WES is the only disease surveillance being conducted. However, it is possible that WES could be used in conjunction with clinical or event-based surveillance. Additionally, we assume that the information from WES is a binary of whether a specific pathogen is present. It is possible that practitioners may want to measure the level of pathogen presence in WES samples, or that a binary detection value can be used to determine which communities’ clinical surveillance should be scaled up. The Centers for Disease Control in the United States, for example, track the relative levels of Sars-CoV-2, influenza, and respiratory syncytial virus in wastewater but only the presence or absence of mpox virus through the National Wastewater Surveillance System [32]. Towards the aim of evaluating trends, in the present study we consider increasing the shutdown threshold, which lends preliminary insight into how the model behavior changes when shutdown does not occur at the first sign of pathogen presence. In the future, we plan to not only simulate more complex patch relationships and intervention strategies but optimize how to combine different surveillance mechanisms, as well. Such work would be of relevance to areas which are resource constrained.

WES itself is subject to limitations. It is not possible to identify which individual(s) are infected, making it impossible to individually target intervention efforts. Moreover, WES samples are evaluated using a variety of methods, including culture-based methods and molecular methods (e.g., polymerase chain reactions and shotgun metagenomics), each with their own strengths and weaknesses [1,33]. A more detailed comparison of different types of WES against clinical surveillance, particularly in the content of antibiotic resistance, are provided by in previous work by Larsson and Flach [33].

Our analysis of a two-patch system allows us to gain intuition as to how sampling frequency and site selection change according to different model parameters, such as the level of interaction between patches. We show that minimizing costs to all subpopulations being surveilled requires coordination and purposeful selection of surveillance sites and sampling frequencies. Complex behavior exhibited by our total cost function indicates that the tradeoff between earlier detection and increased disease burden is not straightforward. By using VOI techniques, we provide a framework to evaluate surveillance information by measuring reduction in expected loss associated with WES against a counterfactual with no surveillance. In the future, more complex and context-specific decision models can help reinforce the value of WES for different pathogens and populations.

## Supporting information

S1 Text Extended methods and results. (PDF)
