# Supplementary material for: Optimal sampling frequency and site selection for wastewater and environmental surveillance of infectious pathogens: A value of information assessment
Source: PLoS Comput Biol. 2025 Jun 25;21(6):e1013190. doi: 10.1371/journal.pcbi.1013190 (PMC12193039; doi:10.1371/journal.pcbi.1013190)
Supplement: S1 Text — (PDF) [file pcbi.1013190.s001.pdf]

S1 Text: Extended Methods and Results

Optimal sampling frequency and site selection for wastewater and environmental surveillance of infectious pathogens: a value of information assessment

Isabella Impalli, Erik Bergland, Chadi M. Saad-Roy, Bryan T. Grenfell, Simon A. Levin, D.G. Joakim Larsson, Ramanan Laxminarayan*

*ramanan@onehealthtrust.org

Contents

Supplementary Figures **2**
- Fig A . . . 2
- Fig B . . . 3
- Fig C . . . 4
- Fig D . . . 5
- Fig E . . . 6
- Fig F . . . 7
- Fig G . . . 8
- Fig H . . . 9
- Fig I . . . 10

Appendix A: Derivation of Disease Dynamics **11**

Appendix B: Minimum of Exponential Random Variables is an Exponential Random Variable **13**

Appendix C: Computing Surveillance Effectiveness s_i **14**

Appendix D: Extended Methods for Value of Information Assessment **16**

Appendix E: Computation of Expected Detection Time and Case Counts **18**

Appendix F: Higher Patch Interaction Leads to Symmetry in the Size of Detected Outbreaks **20**

Appendix G: How Symmetry in the Size of Selected Outbreaks Affects the Total Cost Function **21**

Appendix H: Weak Dependence of Cost on Small Patch Interaction **22**

References **23**

Supplementary Figures

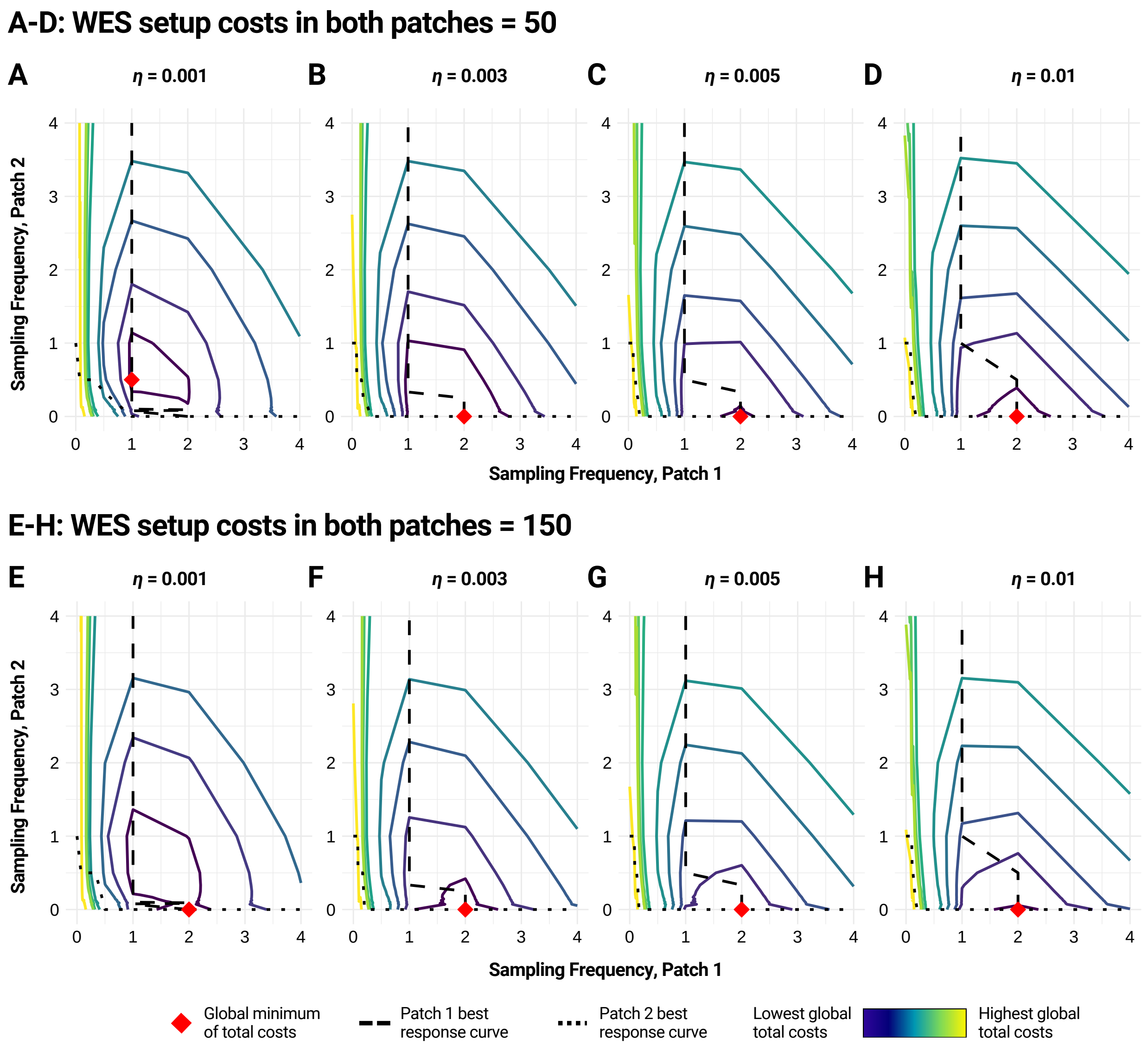

Fig A. Optimal strategies by level of interaction in patches with asymmetric disease dynamics. We illustrate the global total costs (colored gradient) associated with various WES strategies when $k_1 = k_2 = 50$ and A) $\eta = 0.001$, B) $\eta = 0.003$, C) $\eta = 0.005$, and D) $\eta = 0.01$, where $\eta = \eta_{1\to2} = \eta_{2\to1}$, and when $k_1 = k_2 = 150$ and E) $\eta = 0.001$, F) $\eta = 0.003$, G) $\eta = 0.005$, and H) $\eta = 0.01$. The red diamond represents the global minimum of total costs for each parameter set. Best response curves for patch 1 are shown as a dashed black line; best response curves for patch 2 are shown as a dotted black line. Gradients can be compared across all eight panels. Parameter values: patch size = 5000, $\lambda_1 = \lambda_2 = 0.15$, $r_1 = 0.4$, $r_2 = 0.2$, $a_1 = a_2 = 50$, $C_{I,1} = C_{I,2} = 10$. Based on 100,000 simulations and a WES protocol that takes 10 independent samples at specified frequencies (see Methods in Main Text); the gradient in between simulated points is interpolated.

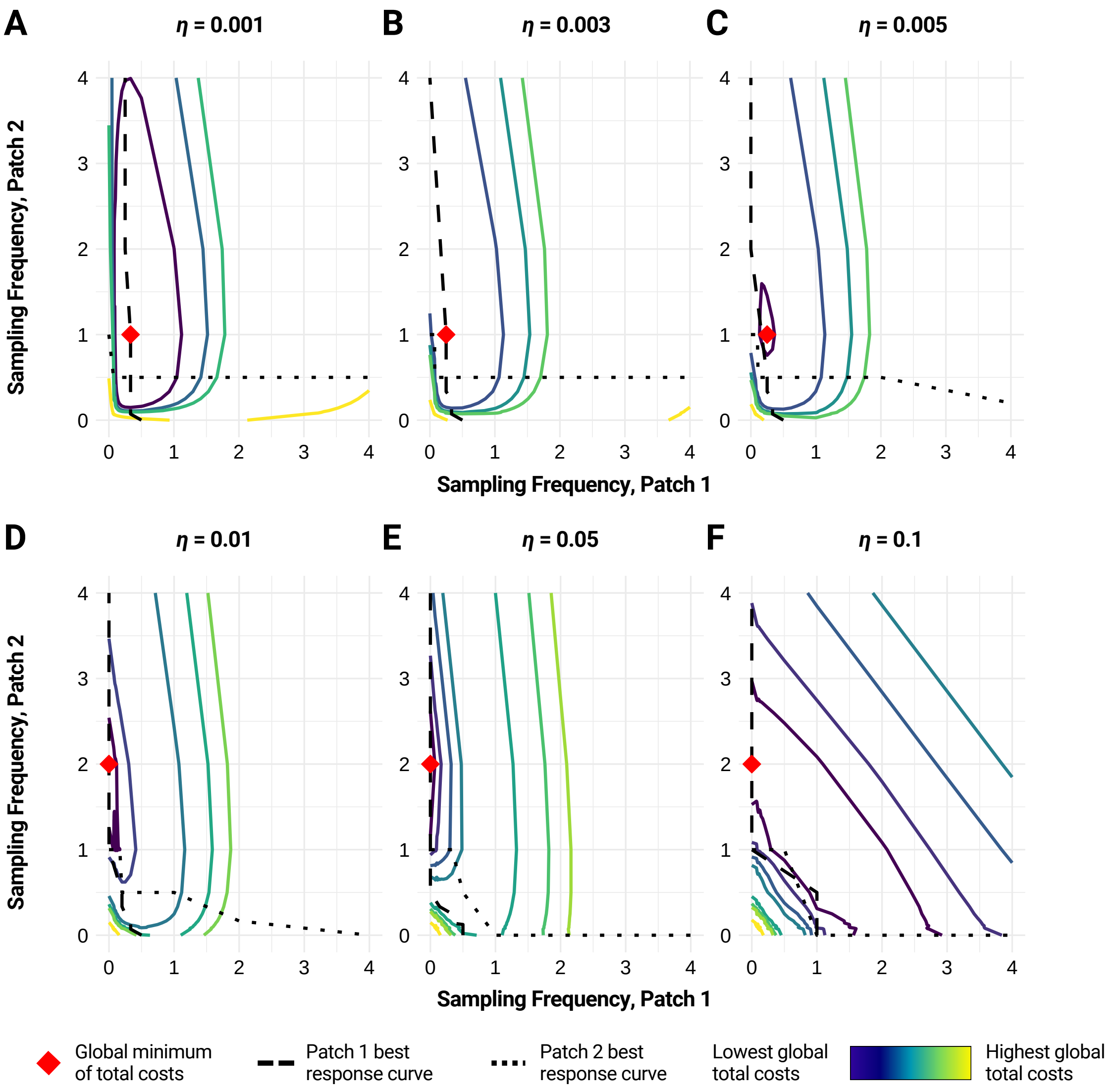

Fig B. Optimal strategies by level of interaction in patches with asymmetric WES costs. We illustrate the global total costs (colored gradient) associated with various WES strategies when A) $\eta = 0.001$, B) $\eta = 0.003$, C) $\eta = 0.005$, D) $\eta = 0.01$, E) $\eta = 0.05$, and F) $\eta = 0.1$, where $\eta = \eta_{1\to2} = \eta_{2\to1}$. The red diamond represents the global minimum of total costs for each parameter set. Best response curves for patch 1 are shown as a dashed black line; best response curves for patch 2 are shown as a dotted black line. Parameter values: patch size $= 5000$, $\lambda_1 = \lambda_2 = 0.15$, $r_1 = r_2 = 0.2$, $k_1 = k_2 = 50$, $a_1 = 200, a_2 = 50$, $C_{I,1} = C_{I,2} = 10$. Based on 100,000 simulations and a WES protocol that takes 10 independent samples at specified frequencies (see Methods in Main Text); the gradient in between simulated points is interpolated.

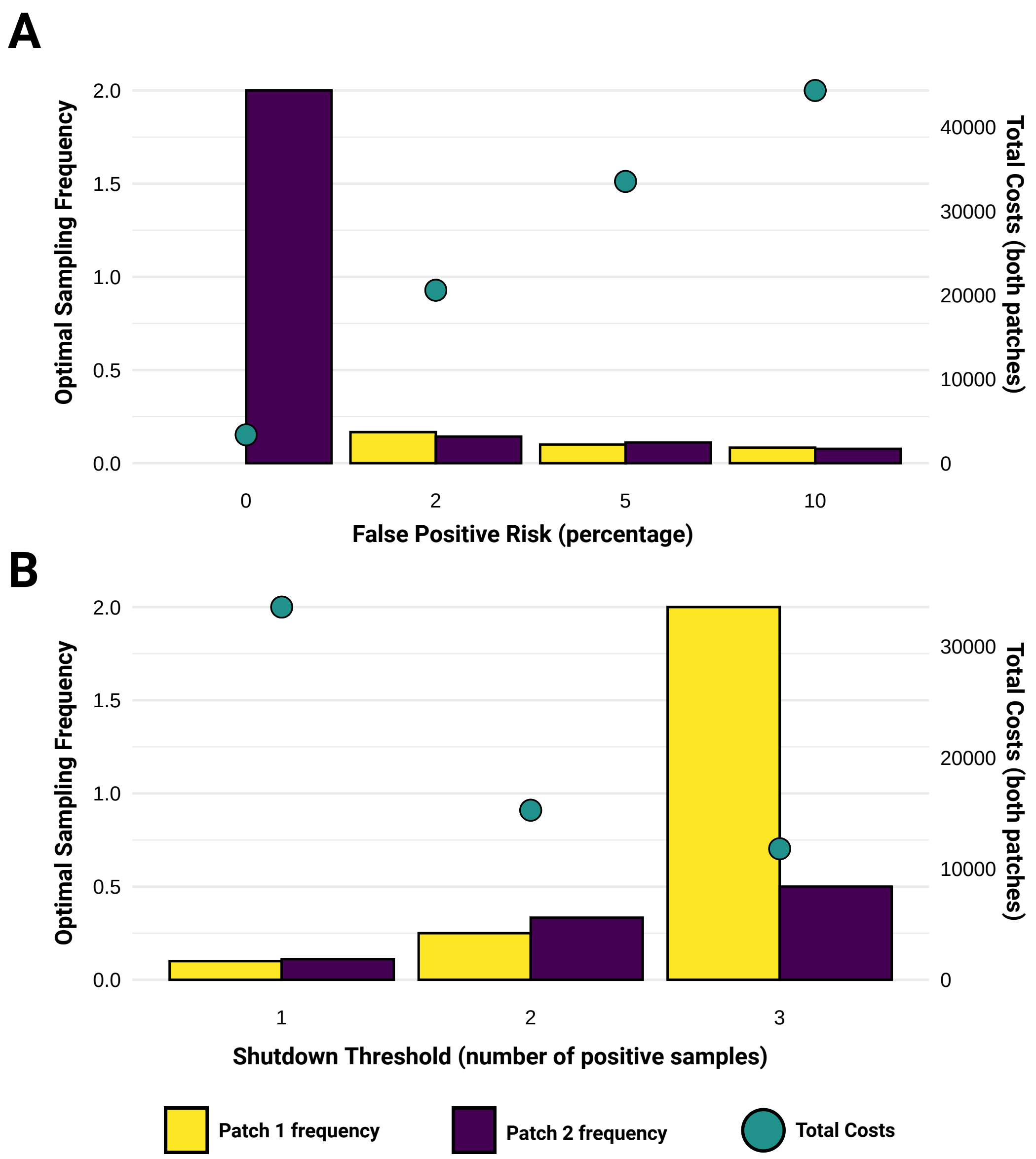

Fig C. Impact of false positivity and differing shutdown thresholds on optimal strategy. We illustrate the optimal sampling frequency in each patch and total costs associated with A) increasing levels of WES false positive risk and B) an increasing shutdown threshold under a constant false positive risk of 2 percent. Other parameter values: patch size = 5000, $\lambda_1 = \lambda_2 = 0.15$, $r_1 = r_2 = 0.2$, $\eta_{2\to1} = \eta_{1\to2} = 0.1$, $k_1 = k_2 = 50$, $a_1 = a_2 = 50$, $C_{I,1} = C_{I,2} = 10$, $C_{S,1} = C_{S,2} = 5$.

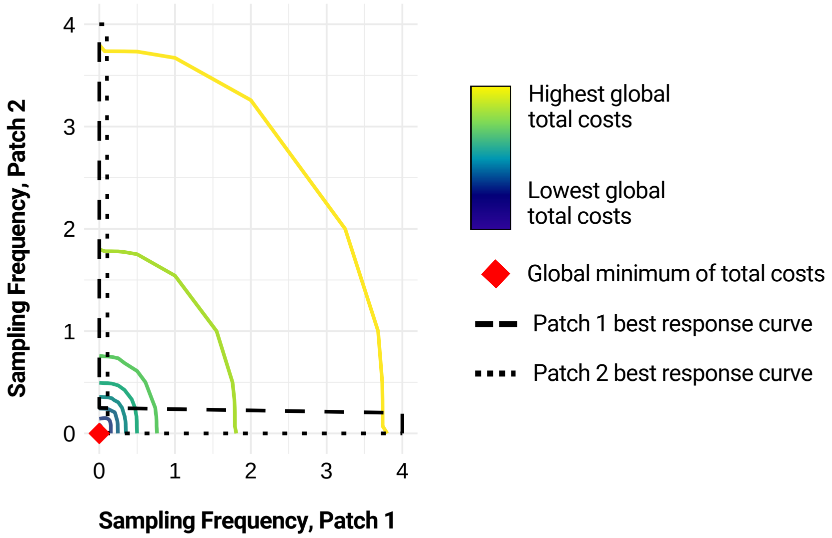

Fig D. Effect of high false positivity and high costs of premature shutdown. We illustrate a scenario with a high false positivity rate (10 percent) and a high cost of premature shutdown ($C_{S,1} = C_{S,2} = 15$). Other parameter values: patch size = 5000, $\lambda_1 = \lambda_2 = 0.15$, $r_1 = 0.4$, $r_2 = 0.2$, $\eta_{2\to1} = \eta_{1\to2} = 0.1$, $k_1 = k_2 = 50$, $a_1 = a_2 = 50$, $C_{I,1} = C_{I,2} = 10$. Based on 100,000 simulations and a WES protocol that takes 10 independent samples at specified frequencies (see Methods in Main Text); the gradient in between simulated points is interpolated.

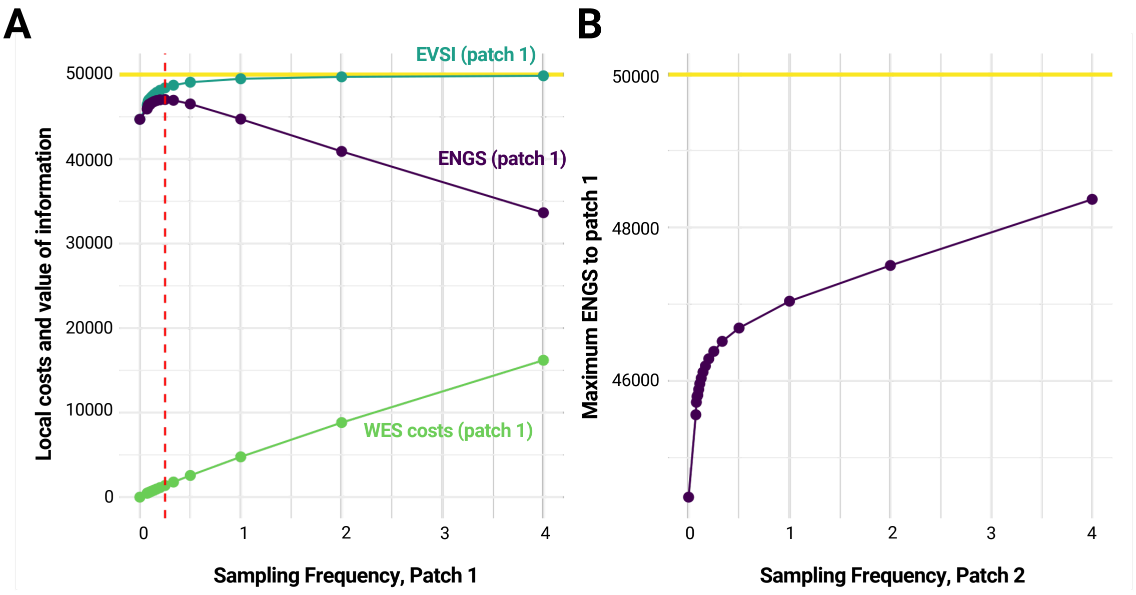

Fig E. Value of information for one patch. We illustrate A) the local wastewater and environmental surveillance (WES) costs, expected value of sample information (EVSI) in patch 1, and expected net gain of sampling (ENGS) in patch 1, given daily sampling in patch 2 and B) the maximum local ENGS for patch 1, given specified WES sampling frequencies in patch 2. The dashed red line indicates the sampling frequency of patch 1 for which ENGS in patch 1 is maximized. The solid yellow line indicates the expected value of perfect information (EVPI) in patch 1. Based on 100,000 simulations and a WES protocol that takes 10 independent samples at specified frequencies with a detection rate dependent on infection burden (see Methods). Data points are connected by straight lines for illustration. Parameter values: patch size = 5000, $\lambda_1 = \lambda_2 = 0.15$, $r_1 = r_2 = 0.2$, $\eta_2 \to 1 = \eta_1 \to 2 = 0.003$, $k_1 = k_2 = 50$, $a_1 = 200, a_2 = 50$, $C_{I,1} = C_{I,2} = 10$, and false positivity rate = 0.

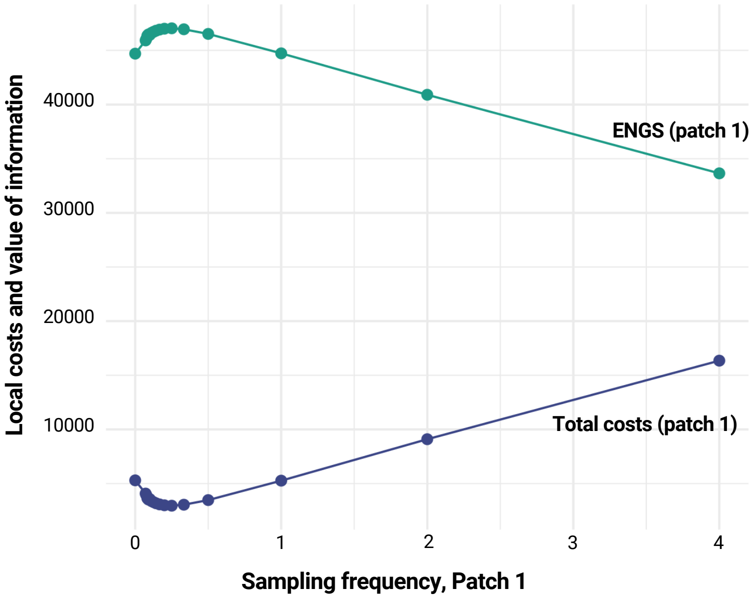

Fig F. Local expected net gain of sampling and total costs. We illustrate the local total costs and expected net gain of sampling (ENGS) for patch 1, given daily sampling in patch 2. Parameter values: patch size = 5000, $\lambda_1 = \lambda_2 = 0.15$, $r_1 = r_2 = 0.2$, $k_1 = k_2 = 50$, $a_1 = 200$, $a_2 = 50$, $C_{I,1} = 5$, $C_{I,2} = 10$, and false positivity rate = 0. Based on 100,000 simulations and a WES protocol that takes 10 independent samples at specified frequencies with a detection rate dependent on infection burden (see Methods in Main Text); straight lines shown between data points for illustration.

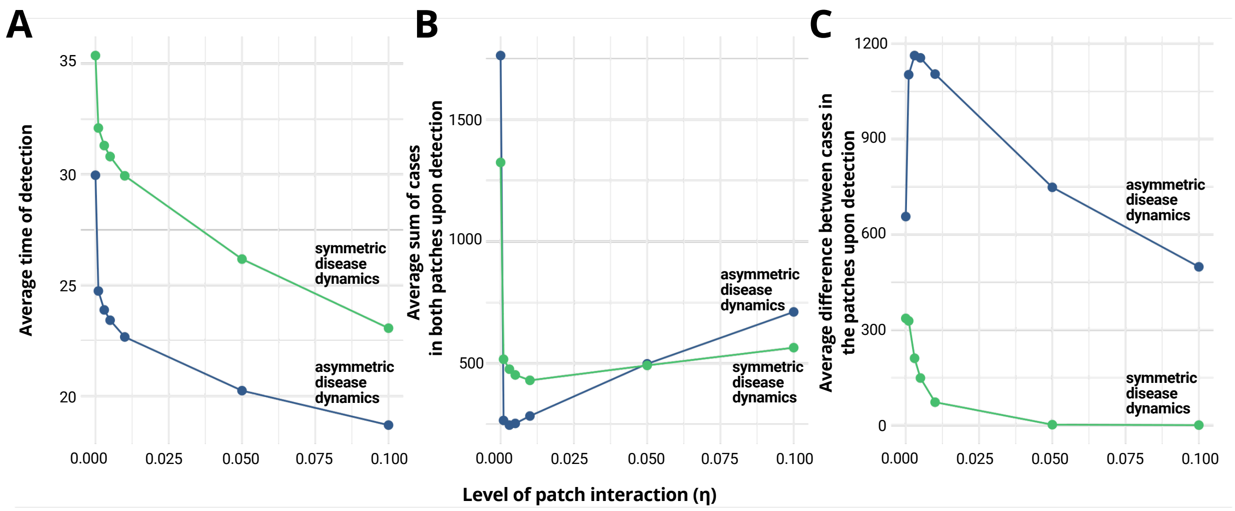

Fig G. Outbreak features by level of interaction. We illustrate the A) average time of detection, B) average sum of cases in both patches upon detection, and C) average of difference between cases in the patches upon detection at varying levels of patch interaction for the case where disease dynamics are symmetric ($r_1 = r_2 = 0.2$) and asymmetric ($r_1 = 0.4$, $r_2 = 0.2$). For panel C), the quantity expressed is the average of the absolute value of the difference between the case counts in both patches upon detection. Other parameter values: patch size = 5000, $\lambda_1 = \lambda_2 = 0.15$, and false positivity rate = 0. Based on 100,000 simulations per parameter set and a WES protocol that takes 10 independent samples at specified frequencies (see Methods in Main Text); straight lines shown between data points for illustration.

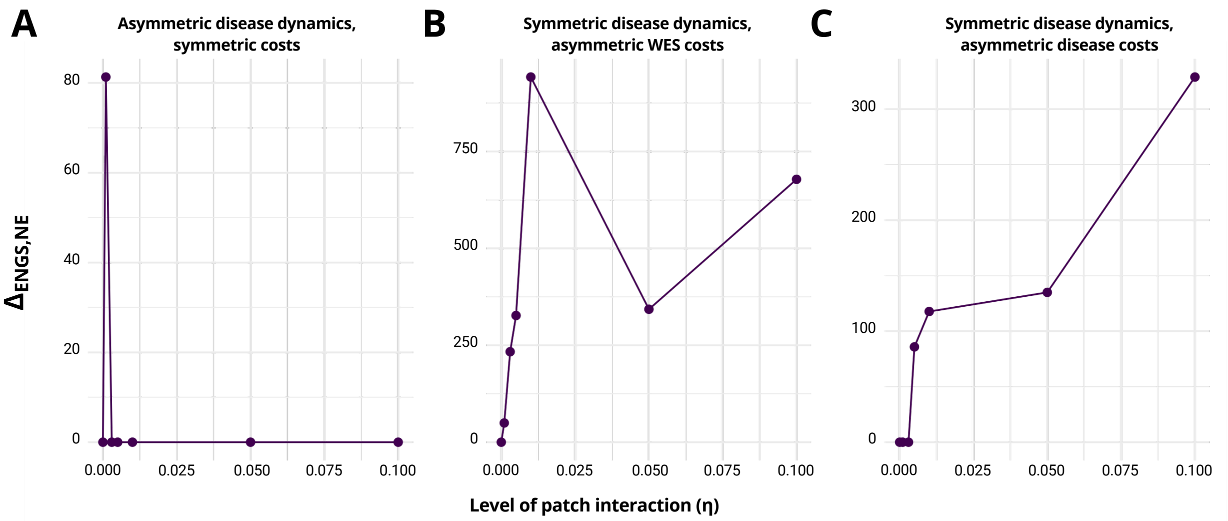

Fig H. Expected net loss associated with suboptimal WES strategies. We illustrate the $\Delta_{ENGS,NE}$, or the difference in the expected net gain of sampling (ENGS) when operating at the globally optimal strategy and operating at the Nash equilibrium, at varying rates of patch interaction under different parameter scenarios: A) asymmetric disease dynamics and symmetric costs, B) symmetric disease dynamics and asymmetric wastewater and environmental surveillance (WES) costs, and C) symmetric disease dynamics and asymmetric disease costs. Parameter values: A) patch size = 5000, $\lambda_1 = \lambda_2 = 0.15$, $r_1 = 0.4$, $r_2 = 0.2$, $k_1 = k_2 = 50$, $a_1 = a_2 = 50$, $C_{I,1} = C_{I,2} = 10$, false positivity rate = 0; B) same as A) except $r_1 = 0.2$ and $a_1 = 200$; C) same as A) except $r_1 = 0.2$ and $C_{I,1} = 5$. Based on 100,000 simulations per parameter set and a WES protocol that takes 10 independent samples at specified frequencies with a detection rate dependent on infection burden (see Methods in Main Text); straight lines shown between data points for illustration.

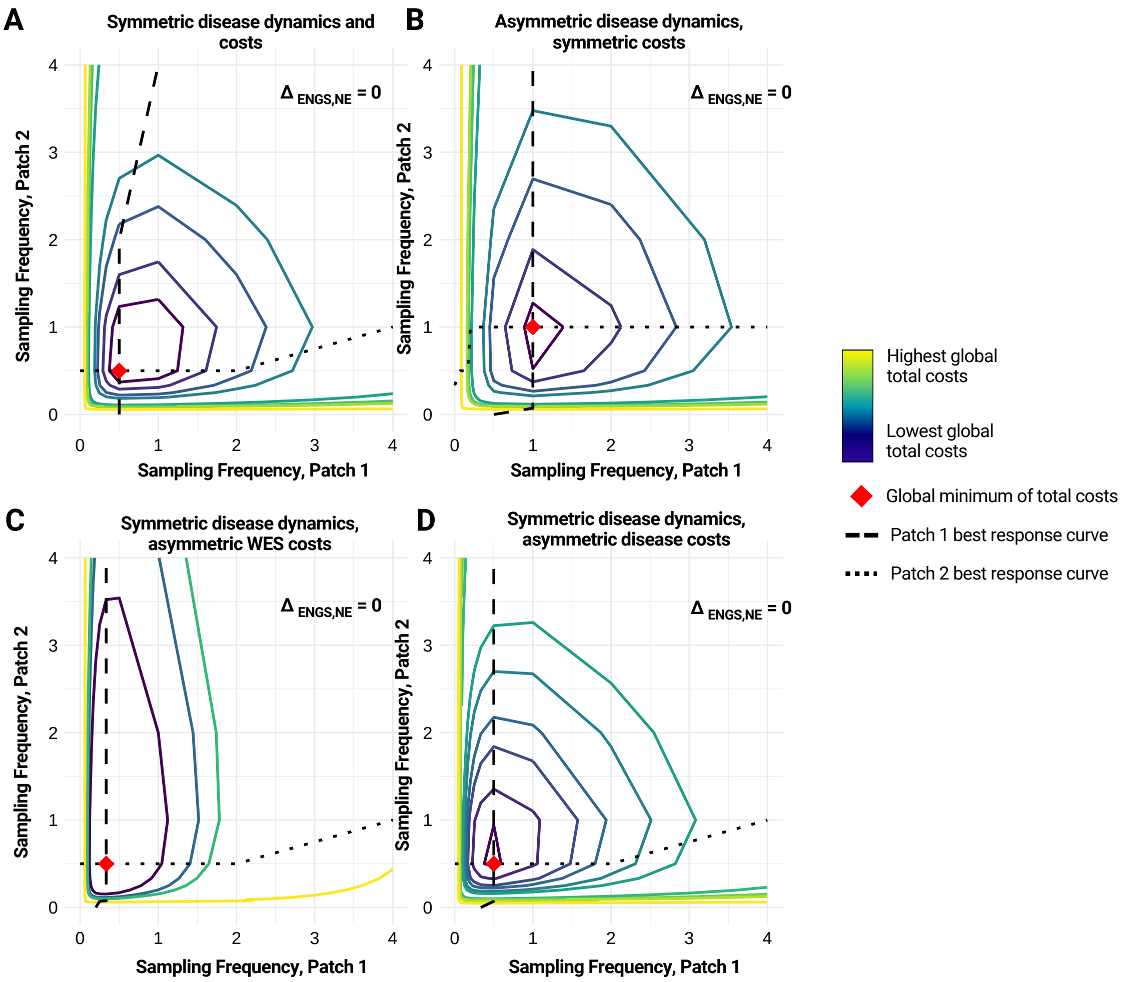

Fig I. Global total costs when patches have uncoupled disease dynamics. We illustrate the global total costs (colored gradient) and indicate the $\Delta_{ENGS,NE}$, or the difference between the expected net gain of sampling (ENGS) at the optimal strategy and at the Nash equilibrium, under different parameter scenarios: A) symmetric disease dynamics and symmetric costs, B) asymmetric disease dynamics and symmetric costs, C) symmetric disease dynamics and asymmetric wastewater and environmental surveillance (WES) costs, and D), symmetric disease dynamics and asymmetric disease costs. The red diamond represents the global minimum of total costs for each parameter set. Best response curves for patch 1 are shown as a dashed black line; best response curves for patch 2 are shown as a dotted black line. Gradients cannot be compared across panels. Parameter values: A) $\lambda_1 = \lambda_2 = 0.15$, $r_1 = r_2 = 0.2$, $k_1 = k_2 = 50$, $a_1 = a_2 = 50$, $C_{I,1} = C_{I,2} = 10$, false positive rate $= 0$; B) same as A) except $r_1 = 0.4$; C) same as A) except $a_1 = 200$; D) same as A) $C_{I,1} = 5$. Based on 100,000 simulations per parameter set and a WES protocol that takes 10 independent samples at specified frequencies (see Methods in Main Text); the gradient in between simulated points is interpolated.

Appendix A: Derivation of Disease Dynamics

We seek to linearize a compartmental model about a disease-free state in order to capture transient disease dynamics between n patches after disease arrives in one of the patches. For illustrative purposes, here we derive the dynamics for $n = 2$ patches.

We begin with an SI model, where the population flows from a susceptible compartment to an infected compartment in each patch i. Within a patch, the rate at which the population flows from susceptible to infected is described by the product of r_i and the current amount of the population which is infected. Here, we use the convention that S_i represents the proportion of the population of patch i (total population $= N_i$) which is susceptible to disease, and I_i is the proportion of the population which is infected.

The flux between compartments in a patch can also be modulated by the infected populations in other patches. Let the product of $\eta_{j\to i}$ and I_j describe the rate at which S_i flows to I_i due to interactions with patch j. The dynamics of a two-patch system are described by

$$\frac{dS_1}{dt} = -r_1 S_1 I_1 - \eta_{2\to 1} S_1 I_2 \tag{1a}$$

$$\frac{dS_2}{dt} = -r_2 S_2 I_2 - \eta_{1\to 2} S_2 I_1 \tag{1b}$$

$$\frac{dI_1}{dt} = r_1 S_1 I_1 + \eta_{2\to 1} S_1 I_2 \tag{1c}$$

$$\frac{dI_2}{dt} = r_2 S_2 I_2 + \eta_{1\to 2} S_2 I_1 \tag{1d}$$

If we linearize the system about a disease free state where $S_i = 1$ and $I_i = 0$ for all i, we find

$$\begin{bmatrix} \dot{S}_1 \\ \dot{S}_2 \\ \dot{I}_1 \\ \dot{I}_2 \end{bmatrix} = \begin{bmatrix} 0 & 0 & -r_1 & -\eta_{2\to 1} \\ 0 & 0 & -\eta_{1\to 2} & -r_2 \\ 0 & 0 & r_1 & \eta_{2\to 1} \\ 0 & 0 & \eta_{1\to 2} & r_2 \end{bmatrix} \begin{bmatrix} S_1 \\ S_2 \\ I_1 \\ I_2 \end{bmatrix} \tag{2}$$

For this analysis, we only care about the dynamics for the infected compartments in the patches. Therefore we simplify to

$$\begin{bmatrix} \dot{I}_1 \\ \dot{I}_2 \end{bmatrix} = \begin{bmatrix} r_1 & \eta_{2\to 1} \\ \eta_{1\to 2} & r_2 \end{bmatrix} \begin{bmatrix} I_1 \\ I_2 \end{bmatrix} \tag{3}$$

We can also examine the dynamics for different model choices. For instance, we may incorporate a mechanism where the infecteds recover at some rate γ_i, and move immediately back to the susceptible compartment (an SIS model). The dynamics of the two-patch system are then described by

$$\frac{dS_1}{dt} = -r_1 S_1 I_1 - \eta_{2\to 1} S_1 I_2 + \gamma_1 I_1 \tag{4a}$$

$$\frac{dS_2}{dt} = -r_2 S_2 I_2 - \eta_{1\to 2} S_2 I_1 + \gamma_2 I_2 \tag{4b}$$

$$\frac{dI_1}{dt} = r_1 S_1 I_1 + \eta_{2\to 1} S_1 I_2 - \gamma_1 I_1 \tag{4c}$$

$$\frac{dI_2}{dt} = r_2 S_2 I_2 + \eta_{1\to 2} S_2 I_1 - \gamma_2 I_2 \tag{4d}$$

And linearizing the system about the disease-free state $(S_i = 1, I_i = 0)$ gives

$$\begin{bmatrix}\dot{S}_1\\ \dot{S}_2\\ \dot{I}_1\\ \dot{I}_2\end{bmatrix} = \begin{bmatrix}0 & 0 & -r_1+\gamma_1 & -\eta_{2\to 1}\\ 0 & 0 & -\eta_{1\to 2} & -r_2+\gamma_2\\ 0 & 0 & r_1-\gamma_1 & \eta_{2\to 1}\\ 0 & 0 & \eta_{1\to 2} & r_2-\gamma_2\end{bmatrix}\begin{bmatrix}S_1\\ S_2\\ I_1\\ I_2\end{bmatrix} \tag{5}$$

To only capture the growth and dynamics among infecteds, we simplify to

$$\begin{bmatrix}\dot{I}_1\\ \dot{I}_2\end{bmatrix} = \begin{bmatrix}r_1-\gamma_1 & \eta_{2\to 1}\\ \eta_{1\to 2} & r_2-\gamma_2\end{bmatrix}\begin{bmatrix}I_1\\ I_2\end{bmatrix} \tag{6}$$

We notice the patch dynamics among infected compartments are similar to the SI model with the inclusion of each patch's recovery term which effectively modulates the growth rate r. Incorporating a third compartment for the recovered population that is no longer susceptible to the disease (SIR), we find the dynamics are described by

$$\frac{dS_1}{dt} = -r_1 S_1 I_1 - \eta_{2\to 1} S_1 I_2 + \delta_1 R_1 \tag{7a}$$
$$\frac{dS_2}{dt} = -r_2 S_2 I_2 - \eta_{1\to 2} S_2 I_1 + \delta_2 R_2 \tag{7b}$$
$$\frac{dI_1}{dt} = r_1 S_1 I_1 + \eta_{2\to 1} S_1 I_2 - \gamma_1 I_1 \tag{7c}$$
$$\frac{dI_2}{dt} = r_2 S_2 I_2 + \eta_{1\to 2} S_2 I_1 - \gamma_2 I_2 \tag{7d}$$
$$\frac{dR_1}{dt} = \gamma_1 I_1 - \delta_1 R_1 \tag{7e}$$
$$\frac{dR_2}{dt} = \gamma_2 I_2 - \delta_2 R_2 \tag{7f}$$

And linearizing the system about the new disease-free state $(S_i = 1, I_i = 0, R_i = 0)$ gives

$$\begin{bmatrix}\dot{S}_1\\ \dot{S}_2\\ \dot{I}_1\\ \dot{I}_2\\ \dot{R}_1\\ \dot{R}_2\end{bmatrix} = \begin{bmatrix}0 & 0 & -r_1 & -\eta_{2\to 1} & \delta_1 & 0\\ 0 & 0 & -\eta_{1\to 2} & -r_2 & 0 & \delta_2\\ 0 & 0 & r_1-\gamma_1 & \eta_{2\to 1} & 0 & 0\\ 0 & 0 & \eta_{1\to 2} & r_2-\gamma_2 & 0 & 0\\ 0 & 0 & \gamma_1 & 0 & -\delta_1 & 0\\ 0 & 0 & 0 & \gamma_2 & 0 & -\delta_2\end{bmatrix}\begin{bmatrix}S_1\\ S_2\\ I_1\\ I_2\\ R_1\\ R_2\end{bmatrix} \tag{8}$$

Among infected compartments, linearized dynamics are identical to the SIS model:

$$\begin{bmatrix}\dot{I}_1\\ \dot{I}_2\end{bmatrix} = \begin{bmatrix}r_1-\gamma_1 & \eta_{2\to 1}\\ \eta_{1\to 2} & r_2-\gamma_2\end{bmatrix}\begin{bmatrix}I_1\\ I_2\end{bmatrix} \tag{9}$$

For ease of illustration, we move forward with the linearized dynamics given directly by the SI model. However, we note that these dynamics could also be derived from an SIS or SIR model, where we implement the effective rate of disease growth $(r_i - \gamma_i)$.

Appendix B: Minimum of Exponential Random Variables is an Exponential Random Variable

Let us suppose that we have n independent exponentially-distributed random variables, $X_i \sim \text{Exp}(\lambda_i)$. We wish to show that $\min\{X_i\}_{i=1}^n$ is also exponentially-distributed. We proceed by induction.

The crux of the argument can be found in [1], which we use to establish the base case. Suppose that we have two independent exponentially-distributed random variables X_1 and X_2 with their accompanying rate parameters. We shall calculate the cumulative distribution function of their minimum. We observe that for $x > 0$,

$$\begin{aligned}\mathbb{P}(\min\{X_1, X_2\} \leq x) &= 1 - \mathbb{P}(\min\{X_1, X_2\} > x) \\ &= 1 - \mathbb{P}(X_1 > x, X_2 > x) \\ &= 1 - \mathbb{P}(X_1 > x)\mathbb{P}(X_2 > x) \\ &= 1 - e^{-\lambda_1 x} e^{-\lambda_2 x} \\ &= 1 - e^{-(\lambda_1 + \lambda_2)x}\end{aligned}$$

where in the third line we have made use of the independence of the variables and in the penultimate we have made use of the (complement of the) cumulative distribution function of exponential random variables. The result is the cumulative distribution function of an exponential random variable with rate parameter $\lambda_1 + \lambda_2$. Therefore, we have established the base case.

Let us now suppose that it is known that the minimum of k exponentially-distributed independent random variables $\{X_i\}_{i=1}^n$ with rate parameters $\{\lambda_i\}_{i=1}^n$ is also exponentially-distributed with rate parameter $\sum_{i=1}^n \lambda_i$. We now suppose that we wish to know the distribution of the minimum of $k+1$ such variables.

We have that

$$\min\{X_i\}_{i=1}^{k+1} = \min\{X_{k+1}, \min\{X_i\}_{i=1}^k\}.$$

Intuitively, this holds since taking the minimum of a collection of numbers is the same as removing one number, computing the minimum of what remains, and then comparing this minimum to the removed number. If the minimum is less than or equal to this number, then it is less than or equal to all of the original numbers. If not, the removed number must be the minimum.

However, we note that $\min\{X_i\}_{i=1}^k$ is an exponentially-distributed random variable with rate parameter $\sum_{i=1}^k \lambda_i$, and X_{k+1} is also exponentially distributed. Furthermore, these variables are independent. Thus, the base case proves that their minimum is exponentially-distributed, and its rate parameter is

$$\lambda_{k+1} + \sum_{i=1}^{k} \lambda_i = \sum_{i=1}^{k+1} \lambda_i =: \lambda$$

as required.

Appendix C: Computing Surveillance Effectiveness s_i

Given that the epidemic begins in patch i, we assume that its detection can be modeled by a Poisson process with rate s_i, which we called the surveillance effectiveness in the main text. In order to determine the surveillance effectiveness for a given initial patch, we suppose that each patch searching for the epidemic has its own surveillance effectiveness, which we denote by $s_i^{(j)}$. We further assume that the effectiveness of patch j is some multiple of the testing frequency f_j. In other words, we have that

$$s_i^{(j)} = d_{ij} f_j \tag{10}$$

for some constant d_{ij}. The detection time of the epidemic is then given by the minimum of the detection time across all patches. In Appendix A, we proved that the minimum of a group of independent exponential random variables is once again an exponential random variable. Furthermore, the rate parameter of the resulting variable is equal to the sum of the rate parameters of the individual variables. Therefore, we find that

$$s_i = \sum_{j=1}^{n} s_i^{(j)} = \sum_{j=1}^{n} d_{ij} f_j \tag{11}$$

The only remaining task is to choose a suitable form for the constant of proportionality d_{ij}. We proceed as follows: first, we imagine that instead of sampling continuously in a given patch with frequency f_j, we carry out surveillance every $\frac{1}{f_j}$ days. One can then ask what the probability for finding the disease on the first day of testing is.

We suppose that this probability is $1 - \varepsilon^{h_{ij}}$, where we refer to ε as the **base miss chance**. For larger values of ε, there is a higher chance the disease is not detected. Similarly, for smaller h_{ij} the failure probability is larger. By utilizing the CDF of an exponential random variable, we note that the probability of detection occurring by day $\frac{1}{f_j}$ can also be written as

$$1 - \epsilon^{h_{ij}} = \mathbb{P}(t_{\text{detect}} \leq \frac{1}{f_j}) = 1 - e^{-d_{ij}}.$$

Rearranging, we find that

$$d_{ij} = -h_{ij} \ln \varepsilon. \tag{12}$$

Therefore, in order to parametrize our model, we set some base rate of missing the epidemic, and then choose a matrix with elements h_{ij} to modify this probability. In order to introduce dependence on the arrival patch, we suppose that the disease arrives in patch i. To approximate how strongly an arrival affects each patch, we compute $A\mathbf{e_i}$, where A is the matrix determined by the right-hand side of **Equation 3**. We then set h_{ij} equal to the jth component of this quantity, normalized by the maximum over all j, so that the largest element of H is one. In other words,

$$h_{ij} = \frac{\mathbf{e_j}^T A \mathbf{e_i}}{\max_{1 \leq k \leq n} \mathbf{e_k}^T A \mathbf{e_i}}. \tag{13}$$

One could replace the matrix A with a number of other candidates in the above formulation. The most obvious choice would be e^{At_0} for some value of t_0. However, one is then left to try to select a value of t_0. For small t_0, one can approximate the matrix exponential by $I + t_0 A$, which de-emphasizes the dependendence of the detection on the actual network structure encoded in A.

Selecting larger values of t_0 would be more appropriate for long detection times. However, for sufficiently long times, linear dynamics are not a satisfactory approximation to the spread of the outbreak. Ultimately due to the additional challenges with the purely theoretical model discussed in the main text and this document, the authors chose to retain the current methodology and defer a more detailed choice to future work.

We note that we have made the somewhat arbitrary choice to normalize the h_{ij} so that they are less than or equal to 1. If this were not the case, let us define $h_{\max}$ to be the largest of the h_{ij}. We may rewrite (12) as

$$d_{ij} = -\frac{h_{ij}}{h_{\max}} \ln \varepsilon^{h_{\max}} \tag{14}$$

using standard rules for logarithms. We can then take $\tilde{h}_{ij} \equiv \frac{h_{ij}}{h_{\max}}$ to be our new h_{ij}, which are by definition less than or equal to 1. Thus, choosing a different normalization is exactly equivalent to the original setup, albeit with a smaller value for the base miss chance.

Appendix D: Extended Methods for Value of Information Assessment

Here, we develop a framework to determine the value of information which allows decision-makers to update their priors on the time of the first disease arrival, t_{arrival}.

The first step in developing the framework is to consider what the prior on t_{arrival} is, and therefore, the decision made under current information. Our setup discussed in the Main Text assumes that in the absence of surveillance information (i.e., current information), the patches do not shut down until the disease spreads throughout both patches. The expected loss of this decision is always $\sum_i C_{I,i} N_i$ for all patches i, as the decision-maker effectively assumes that the disease is never expected to arrive.

We may consider another scenario where the decision-maker has knowledge of the value of λ_{tot}, and therefore the default decision under 'current information' is to shut down the patches at $\mathbb{E}(t_{\text{arrival}}) = 1/\lambda_{\text{tot}}$. In this scenario, however, we have the potential to incur costs from premature shutdowns or from shutdowns which occur late, when infections have started spreading. The probability of at least 1 disease arrival by $t = 1/\lambda_{\text{tot}}$ is $1 - 1/e$, making the probability of premature shutdown $1/e$. The expected number of infections at $t = 1/\lambda_{\text{tot}}$ can be determined numerically following the growth dynamics described in **Appendix A**.

We next want to determine the value of perfect surveillance, which pinpoints t_{arrival} every single time, thereby limiting the costs incurred to a single infection: $\sum_i \frac{\lambda_i}{\lambda_{\text{tot}}} \cdot C_{I,i}$. For the model discussed in the Main Text, where the default decision is to let infection spread throughout both patches, we define the expected value of perfect information (EVPI) as

$$\text{EVPI} = \sum_i C_{I,i} N_i - \sum_i \frac{\lambda_i}{\lambda_{\text{tot}}} \cdot C_{I,i} = \sum_i C_{I,i} (N_i - \frac{\lambda_i}{\lambda_{\text{tot}}}) \tag{15}$$

where $C_{I,i}$ is the cost associated with each infection, N_i is the population of patch i, λ_i is the arrival rate of new infections in patch i, λ_{tot} is the sum of arrival rates across all patches i, and the counterfactual scenario is no WES program (which means that there is no patch shutdown, as discussed above).

For the second model which assumes a default policy which shuts down the patches at $\mathbb{E}(t_{\text{arrival}}) = 1/\lambda_{\text{tot}}$, we replace the first term of the equation with the actual costs incurred from this policy.

We also note that in this analysis, the EVPI is equivalent to the expected value of perfect parameter information (EVPPI). EVPPI is used to determine the value of resolving uncertainty around single parameters or parameter sets. Even though, in reality, information can rarely be "perfect," EVPI and EVPPI are used as a checkpoint to determine the upper threshold of information value for all parameters or a given parameter set, respectively. In our simplified decision model, there is only one parameter of interest: whether the surveillance detects an infection (which may or may not reflect the true state of the system), which acts as the trigger to make the decision to 'shut down' the patches. Having only one parameter of interest allows us to equate the EVPI and the EVPPI, though we note their theoretic distinction.

Similarly, we define the expected value of sample information (EVSI), which is specific to some surveillance strategy x, as:

$$\begin{aligned}\text{EVSI}_x = \sum_i C_{I,i} N_i - \sum_i C_{I,i} \mathbb{E}[I_{\text{detection},i}]_x - \sum_i \mathbb{P}(\text{false detection})_x C_{S,i} N_i = \\ \sum_i C_{I,i} (N_i - \mathbb{E}[I_{\text{detection},i}]_x) - \mathbb{P}(\text{false detection})_x C_{S,i} N_i\end{aligned} \tag{16}$$

where EVSI_x is the expected value of sample information associated with strategy x, N_i is the population of patch i, $C_{I,i}$ is the cost associated with each infection, $C_{S,i}$ is the per capita cost associated with premature patch shutdown, $I_{\text{detection},i}$ is the size of the outbreak upon detection under strategy x in patch i, $\mathbb{P}$(false detection) is the overall probability of false detection in all patches, and the counterfactual is no

WES program. $\mathbb{E}(I_{\text{detection},i})_x$ and $\mathbb{P}(\text{false detection})_x$ must be calculated numerically through simulations for each strategy x.

Appendix E: Computation of Expected Detection Time and Case Counts

We begin by computing the probability that a false detection event occurs. In each patch, false detection events are exponentially distributed with rate parameter $\rho_j f_j$. In order for a false detection to occur for a given outbreak, at least one patch must incorrectly announce a detection prior to the true outbreak of the disease. Therefore, a false detection occurs when the minimum of the times before a patch announces an incorrect detection is less than the time before the arrival of the disease. Denoting the time when a false detection would be announced in patch j by $t_{\text{false},j}$ and the time when the outbreak occurs as t_{arrive}, we have that the probability of a false detection is $\mathbb{P}(\min_j\{t_{\text{false},j}\} \leq t_{\text{arrive}})$.

We recall that the arrival times in each patch i are exponential random variables with rate parameters λ_i, and that the arrival time for the outbreak is the minimum of the times for the two patches. By Appendix B, we can therefore say that a false detection is an exponential random variable with rate parameter $\sum_{j=1}^n \rho_j f_j$ and the arrival time is exponentially distributed with rate parameter λ_{tot}. We therefore obtain

$$\begin{aligned}\mathbb{P}(\min_j\{t_{\text{false},j}\} \leq t_{\text{arrive}}) &= \int_0^\infty \lambda_{\text{tot}} e^{-\lambda_{\text{tot}} u}(1 - e^{-(\sum_{j=1}^n \rho_j f_j)u})\mathrm{d}u \\ &= \frac{\sum_{j=1}^n \rho_j f_j}{\lambda_{\text{tot}} + \sum_{j=1}^n \rho_j f_j}.\end{aligned} \tag{17}$$

Similarly, the probability of a legitimate detection is

$$\mathbb{P}(\min_j\{t_{\text{false},j}\} \geq t_{\text{arrive}}) = 1 - \mathbb{P}(\min_j\{t_{\text{false},j}\} \leq t_{\text{arrive}}) = \frac{\lambda_{\text{tot}}}{\lambda_{\text{tot}} + \sum_{j=1}^n \rho_j f_j}. \tag{18}$$

We now turn to computing the expected detection time. There are two cases. First, in the event of a true detection, we note that as above the arrival time of the otbreak is an exponential random variable, as is the difference between arrival time and detection. For a disease arriving in patch i, the rate parameter of detection is s_i. Making use of the linearity of expectation and the law of total expectation, we have that

$$\begin{aligned}\mathbb{E}[t_{\text{detect}}|\text{true detection}] &= \mathbb{E}[t_{\text{arrive}}|\text{true detection}] + \mathbb{E}[t_{\text{detect}} - t_{\text{arrive}}|\text{true detection}] \\ &= \mathbb{E}[\mathbb{E}[t_{\text{arrive}}|\text{true detection, arrival in patch } i]] \\ &\quad + \mathbb{E}[\mathbb{E}[t_{\text{detect}} - t_{\text{arrive}}|\text{true detection, arrival in patch } i]] \\ &= \mathbb{E}[\frac{1}{\lambda_i}] + \mathbb{E}[\frac{1}{s_i}] \\ &= \frac{n}{\lambda_{\text{tot}}} + \sum_{i=1}^n \frac{\lambda_i}{\lambda_{\text{tot}} s_i}\end{aligned} \tag{19}$$

where in the third line we have used that the mean of an exponential random variable is the inverse of its rate parameter. The final line follows because the probability of arrival in each patch is $\frac{\lambda_i}{\lambda_{\text{tot}}}$. In the second case, the detection event is a false positive. As discussed above, this event is exponentially distributed with rate parameter $\sum_{j=1}^n \rho_j f_j$. Therefore, the expected value of the time of detection in this case is $\frac{1}{\sum_{j=1}^n \rho_j f_j}$.

When the rate of false positives is zero ($\rho_1 = \rho_2 = 0$), only the first case is possible and the expected detection time is given by (19). We note that in this situation, the expected time until a false positive occurs is infinite but has zero probability of occurring prior to the actual arrival of the disease, which necessitates breaking things down by cases. Thus the expected time of detection is

$$\begin{aligned}\mathbb{E}[t_{\text{detect}}] &= \frac{1}{\lambda_{\text{tot}} + \sum_{j=1}^n \rho_j f_j}\Big(n + \sum_{i=1}^n \frac{\lambda_i}{s_i}\Big) + \frac{1}{\lambda_{\text{tot}} + \sum_{j=1}^n \rho_j f_j}\mathbb{1}_{\{\rho_1>0\}\cup\{\rho_2>0\}} \\ &= \frac{1}{\lambda_{\text{tot}} + \sum_{j=1}^n \rho_j f_j}\Big(n + \mathbb{1}_{\cup_{i=1}^n\{\rho_i>0\}} + \sum_{i=1}^n \frac{\lambda_i}{s_i}\Big)\end{aligned} \tag{20}$$

which is simply the weighted sum of the true detection and spurious detection case, with the weight determined by (17).

Let us now turn to the expected case counts given that a detection has occurred. We note that one can explicitly solve for the number of infected individuals at time t, given that the disease arrives at time t_{arrive}. We shall denote the vector containing the initial number of infected individuals by κ. This vector is equal to one of the standard basis vectors $\mathbf{e_i}$ with probability $\frac{\lambda_i}{\lambda_{\text{tot}}}$, representing an arrival in patch i. Rewriting **Equation 3** of the main paper in matrix form, we see that we must solve

$$\begin{aligned} \frac{dI}{dt} &= AI, \quad t \geq t_{\text{arrive}} \\ I(t_{\text{arrive}}) &= \kappa. \end{aligned} \tag{21}$$

This yields

$$I(t) = e^{A(t-t_{\text{arrive}})}\kappa \mathbb{1}_{\{t \geq t_{\text{arrive}}\}} \tag{22}$$

Once again, there are two cases. If an outbreak is falsely detected, then the number of patients at detection is zero. Therefore, the expected number of patients is given by multiplying the expected number of patients under a legitimate detection by the probability of such a detection occurring. Proceeding once more by the law of total expectation, we find that

$$\begin{aligned} \mathbb{E}[e^{A(t_{\text{detect}}-t_{\text{arrive}})}\kappa \mathbb{1}_{\{t_{\text{detect}} \geq t_{\text{arrive}}\}}|\text{true detection}] &= \mathbb{E}[\mathbb{E}[e^{A(t_{\text{detect}}-t_{\text{arrive}})}\kappa \mathbb{1}_{\{t_{\text{detect}} \geq t_{\text{arrive}}\}}|\text{true det., arrive in patch } i \text{ at } t_{\text{arrive}}]] \\ &= \mathbb{E}[\Big(\int_0^\infty s_i e^{Au} e^{-s_i u} \mathrm{d}u\Big)\mathbf{e_i}] \end{aligned}$$

We now assume that for any eigenvalue μ of A, it is necessarily the case that $s_i > \mu$. This enforces a minimum viable testing rate, and ensures that the inner integral converges. With this assumption in hand, we find that

$$\begin{aligned} \mathbb{E}[e^{A(t_{\text{detect}}-t_{\text{arrive}})}\kappa \mathbb{1}_{\{t_{\text{detect}} \geq t_{\text{arrive}}\}}|\text{true detection}] &= \mathbb{E}[\Big(Id - \frac{A}{s_i}\Big)^{-1}\mathbf{e_i}] \\ &= \sum_{i=1}^{n} \frac{\lambda_i}{\lambda_{\text{tot}}}\Big(Id - \frac{A}{s_i}\Big)^{-1}\mathbf{e_i} \end{aligned} \tag{23}$$

Examining the equation above, we observe that as s_i draws closer to any of the eigenvalues of A from above, the expected size of the epidemic becomes infinite, which enforces a minimal amount of testing on the patches as discussed in the main text. For smaller values of s_i, the integral fails to converge. We observe that it is not necessary to make this restriction on any s_i such that $e^{At}\mathbf{e_i}$ is identically zero, since then the integrand is zero as well. We also note that in this framework, we have dispensed with all mentions of the size of the population in the patches.

Multiplying the results of our calculation by the probability of a legitimate detection, we find that

$$\mathbb{E}[e^{A(t_{\text{detect}}-t_{\text{arrive}})}\kappa \mathbb{1}_{\{t_{\text{detect}} \geq t_{\text{arrive}}\}}] = \sum_{i=1}^{n} \frac{\lambda_i}{\lambda_{\text{tot}} + \sum_{j=1}^{n} \rho_j f_j}\Big(Id - \frac{A}{s_i}\Big)^{-1}\mathbf{e_i}. \tag{24}$$

In the case where false positives do not occur, this result correctly reduces to (23).

Appendix F: Higher Patch Interaction Leads to Symmetry in the Size of Detected Outbreaks

In the main text, we have observed that higher levels of interaction between the patches lead to symmetric patch sizes upon detection, given symmetric disease dynamics. We now explore this phenomenon analytically.

To begin, let us consider the linear dynamics preceeding detection. Infections grow according to the linear equation $\dot{I} = AI$. Given that the growth rates and interaction strengths are the same for both patches, A takes the form

$$A = \begin{pmatrix} r & \eta \\ \eta & r \end{pmatrix} \tag{25}$$

with $r, \eta > 0$. Let us now change to polar coordinates, so that

$$I = \rho \begin{pmatrix} \cos(\theta) \\ \sin(\theta) \end{pmatrix}. \tag{26}$$

We note that we must make the restriction $0 \leq \theta \leq \frac{\pi}{2}$ so that the entries of I take only positive values, since they represent case counts. Substituting into the linear ODE, we find that

$$\dot{\rho} \begin{pmatrix} \cos(\theta) \\ \sin(\theta) \end{pmatrix} + \rho\dot{\theta} \begin{pmatrix} -\sin(\theta) \\ \cos(\theta) \end{pmatrix} = \rho \begin{pmatrix} r & \eta \\ \eta & r \end{pmatrix} \begin{pmatrix} \cos(\theta) \\ \sin(\theta) \end{pmatrix}. \tag{27}$$

We now observe that the two vectors on the left-hand side of Equation 27 are perpendicular. Therefore, taking the dot product of both sides of the equation with the second of these vectors yields

$$\rho\dot{\theta} \begin{pmatrix} -\sin(\theta) \\ \cos(\theta) \end{pmatrix} \cdot \begin{pmatrix} -\sin(\theta) \\ \cos(\theta) \end{pmatrix} = \rho \begin{pmatrix} -\sin(\theta) \\ \cos(\theta) \end{pmatrix} \cdot \left[\begin{pmatrix} r & \eta \\ \eta & r \end{pmatrix} \begin{pmatrix} \cos(\theta) \\ \sin(\theta) \end{pmatrix} \right].$$

we can simplify both sides by canceling the common value of ρ and noting that the dot product of our chosen vector with itself is 1. Thus, we obtain

$$\dot{\theta} = \begin{pmatrix} -\sin(\theta) \\ \cos(\theta) \end{pmatrix} \cdot \left[\begin{pmatrix} r & \eta \\ \eta & r \end{pmatrix} \begin{pmatrix} \cos(\theta) \\ \sin(\theta) \end{pmatrix} \right].$$

Finally, let us explicitly simplify the right-hand side. This yields

$$\dot{\theta} = \eta(\cos^2(\theta) - \sin^2(\theta)) = \eta\cos(2\theta). \tag{28}$$

Carrying out similar manipulations after taking the dot product with the first vector on both sides of Equation 27, we arrive at

$$\dot{\rho} = (r + \eta\sin(2\theta))\rho. \tag{29}$$

We immediately note that while the angle θ affects the evolution of the magnitude ρ, the reverse is not true. Since ρ is the magnitude of the vector I, its size determines the total disease burden in the system, while θ determines how these cases are apportioned between the patches. Therefore, we note that the proportions of cases in each patch is determined completely independently from the overall disease burden.

Seeking fixed points of Equation 28, we find that the only value of θ in the interval $[0, \frac{\pi}{2}]$ that suffices is $\theta^* = \frac{\pi}{4}$. To determine whether this fixed point is stable, we take the derivative of the right-hand side of Equation 28 and set $\theta = \frac{\pi}{4}$, yielding

$$\eta \frac{d\cos(2\theta)}{d\theta}\Big|_{\theta=\frac{\pi}{4}} = -2\eta\sin(\frac{\pi}{2}) = -2\eta < 0 \tag{30}$$

Therefore, our fixed point is stable. Since $\cos(\frac{\pi}{4}) = \sin(\frac{\pi}{4})$, we note that this fixed point indicates that the system naturally evolves towards sharing the disease burden equally between the two patches.

Finally, we note that the rate at which the system evolves towards sharing the disease burden is directly proportional to the interaction strength η. It is this fact which explains why high interaction yields patch sizes that are nearly equal upon detection. Whenever a system exhibits symmetric dynamics, if it evolves uninterrupted it will eventually distribute its case load nearly evenly. However, if η is small, detection of the disease happens on a faster timescale, and the symmetry is broken. For larger values of η, the alignment of case counts happens prior to detection.

Appendix G: How Symmetry in the Size of Selected Outbreaks Affects the Total Cost Function

For simplicity, we consider the case where false positives do not occur. Given the symmetry in the size of detected outbreaks in cases of high patch interaction, we seek to understand how this may affect the total cost function.

Recall the total cost of a surveillance program in two patches as simulated in the Main Text is given by

$$TC = k_1 \mathbb{1}_{f_1>0} + a_1 f_1 \mathbb{E}[t_{detection}] + \mathbb{E}[C_{I,1} I_{detection,1}] + k_2 \mathbb{1}_{f_2>0} + a_2 f_2 t_{detection} + C_{I,2}\mathbb{E}[I_{detection,2}] \quad (31)$$

In cases of symmetric outbreak size upon detection, $\mathbb{E}[I_{detection,1}] = \mathbb{E}[I_{detection,2}] = \mathbb{E}[I_{detection}]$. Substituting this quantity and rearranging yields

$$TC = \mathbb{E}[I_{detection}](C_{I,1} + C_{I,2}) + \mathbb{E}[t_{detection}](a_1 f_1 + a_2 f_2) + k_1 \mathbb{1}_{f_1>0} + k_2 \mathbb{1}_{f_2>0} \quad (32)$$

Provided that WES costs are asymmetric (i.e., $a_1 \neq a_2$ or $k_1 \neq k_2$) we see that $TC(f_1, f_2) \neq TC(f_2, f_1)$ in general. However, in cases where WES costs are symmetric (i.e., $a_1 = a_2 = a$, $k_1 = k_2 = k$), then

$$TC = \mathbb{E}[I_{detection}](C_{I,1} + C_{I,2}) + \mathbb{E}[t_{detection}]a(f_1 + f_2) + k\mathbb{1}_{f_1>0} + k\mathbb{1}_{f_2>0} \quad (33)$$

Therefore, we see that $TC(f_1, f_2) = TC(f_2, f_1)$ for any f_1 and f_2 since swapping the frequencies in a symmetric outbreak does not affect the average time of detection. This holds irrespective of whether $C_{I,1} = C_{I,2}$. We note that even with strong interaction, it is only approximately true that $I_{detection,1} = I_{detection,2}$ for any given realization, and thus for any set of simulations the effects of noise will introduce a small difference between the average values. Ultimately, the costs due to choosing frequencies (f_1, f_2) should be very close to that obtained from choosing (f_2, f_1), as seen in **Fig 3** of the Main Text.

Appendix H: Weak Dependence of Cost on Small Patch Interaction

For simplicity, we again consider the case when false positives do not occur. We recall that the total cost of a surveillance program is given by

$$TC = \Big(a \cdot \mathbf{f}\Big)\Big(\frac{n}{\lambda_{\text{tot}}} + \sum_{i=1}^{n} \frac{\lambda_i}{s_i \lambda_{\text{tot}}}\Big) + C_I \cdot \Big(\sum_{i=1}^{n} \frac{\lambda_i}{\lambda_{\text{tot}}} \Big(Id - \frac{A}{s_i}\Big)^{-1} \mathbf{e_i}\Big) + k \cdot \mathbb{1}_{\{\mathbf{f}>0\}} \tag{34}$$

We note that in most of the cases studied throughout this paper, the couplings between patches are at least an order of magnitude smaller than the in-patch growth rates. As such, we might rewrite our growth matrix A as

$$A = A^{(0)} + \varepsilon A^{(1)}$$

where $\varepsilon > 0$ is a small positive number and $A^{(0)}$ is a diagonal matrix. We can similarly write our testing frequencies as

$$f_i = f_i^{(0)} + \varepsilon f_i^{(1)},$$

which would imply a similar expansion for the effectiveness rates s_i. We note that this holds even if the dependence of the effectiveness on the testing frequencies is more complicated than what was given in Appendix B; any smooth function would suffice. Taking $k \equiv 0$ for simplicity, we expand our cost function in a Taylor series around $\varepsilon = 0$ to obtain

$$TC = \Big(a \cdot \mathbf{f^{(0)}}\Big)\Big(\frac{n}{\lambda_{\text{tot}}} + \sum_{i=1}^{n} \frac{\lambda_i}{s_i^{(0)} \lambda_{\text{tot}}}\Big) + C_I \cdot \Big(\sum_{i=1}^{n} \frac{\lambda_i}{\lambda_{\text{tot}}} \Big(Id - \frac{A^{(0)}}{s_i^{(0)}}\Big)^{-1} \mathbf{e_i}\Big) + \mathcal{O}(\varepsilon), \tag{35}$$

where the final term collects all of the terms that are proportional to ε. In the case when $\varepsilon = 0$, only the remaining terms matter, which is equivalent to analyzing the dynamics without coupling between the patches. However, we note that even when the patches are coupled, the last term is much smaller than those corresponding to the uncoupled case. Therefore, the corresponding strategy may remain close to that of the uncoupled case (found by choosing the values of $f_i^{(0)}$ that minimize the first term), while the value of the cost function at the optimal strategy changes slightly depending on the costs of various components of the surveillance program.

For nonzero k, the system could respond discontinuously to small perturbations to frequencies. However, if the optimal sampling strategy of the uncoupled problem is to test in both patches, then for small enough ε the values of the indicator functions will not change, and no discontinuous jump in sampling frequencies will occur.

References

[1] Ewan Delanoy (https://math.stackexchange.com/users/15381/ewan-delanoy). *How to prove that minimum of two exponential random variables is another exponential random variable?* Mathematics Stack Exchange. URL:https://math.stackexchange.com/q/580307 (version: 2013-11-25). eprint: https://math.stackexchange.com/q/580307. URL: https://math.stackexchange.com/q/580307.
